# Thermally Activated Vaporization of Fluorocarbon-In-Hydrocarbon Exoskeletal Droplets

**DOI:** 10.1021/acsomega.5c11095

**Published:** 2026-05-06

**Authors:** William N. Frantz, Awaneesh Upadhyay, Mark A. Borden

**Affiliations:** † Biomedical Engineering Program, 1877 University of Colorado, Boulder, Colorado 80309, United States; ‡ Department of Mechanical Engineering, University of Colorado, Boulder, Colorado 80309, United States

## Abstract

Prior research has demonstrated synthesis and vaporization of hydrocarbon (HC)-in-fluorocarbon (H/F/W) endoskeletal droplets in aqueous suspension through the use of a fluorosurfactant and their potential utility as photoacoustic contrast agents. Here, we describe the synthesis of novel fluorocarbon-in-hydrocarbon (F/H/W) exoskeletal droplets through the choice of phospholipid surfactant. Exoskeletal drops tended to form nonspherical structures due to the elasticity of the solid HC shell. Vaporization of exoskeletal droplets occurred over a longer time scale than endoskeletal droplets due to constricted vapor nucleation and growth, and the resulting bubbles were significantly smaller. The size distribution of the exoskeletal droplets and the resulting bubbles could be further reduced through filtration. In contrast to endoskeletal droplets, which rapidly vaporized from a single nucleation site, exoskeletal droplets formed multiple nucleation sites during vaporization. Heat treatment with quenching increased the number of nucleation sites on each vaporizing droplet. The improved biocompatibility and ability to control the process of vaporization for exoskeletal droplets may be advantageous for medical imaging and other applications.

## Graphical abstract

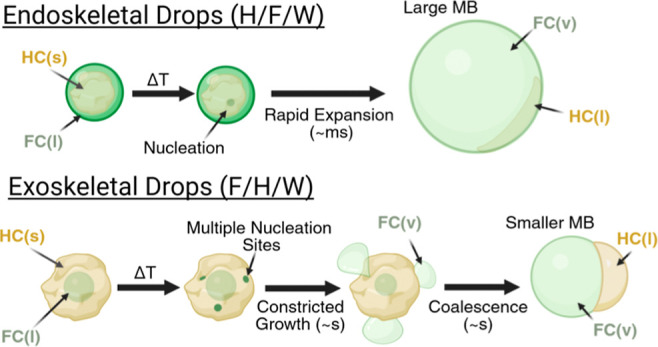

## Introduction

Phase-change droplets are liquid emulsion particles suspended in an aqueous medium that vaporize into microbubbles upon stimulation with applied energy,^1,2^ such as heating,^3^ ultrasound insonation^4^ and exposure to optical^5,6^ or high-energy radiation (e.g., protons, neutrons or X-rays).^7–11^ The resulting microbubble provides optical and acoustic contrast for detection and imaging. For example, phase-change nanodrops have gained interest for biomedical applications in contrast-enhanced ultrasound imaging,^12–14^ radiation detection,^7,8,15^ targeted drug delivery^16–18^ and other diagnostic and therapeutic applications.^19–22^

For ultrasound imaging and therapy, superheated fluorocarbons (FCs), such as perfluoropropane (*n*-C_3_F_8_) and perfluorobutane (*n*-C_4_F_10_), have been used due to their low acoustic thresholds for vaporization.^23,24^ However, the thermodynamic instability of superheated droplets poses risks of colloidal instability and vessel occlusion due to premature vaporization and ripening phenomena.^1,25^ For example, exposure of superheated nanodrops to a gas bubble was shown to induce mass transfer of FC from the liquid droplets to the bubble, a phenomenon observed as “bubble inflation”.^25^ This diffusion process is driven by the vapor pressure gradient between the droplets (vapor pressure of ∼12 bar for C_3_F_8_ and ∼3.9 bar for C_4_F_10_ at 37 °C)^26^ and ambient pressure (∼1 bar) of the gas phase. In principle, once a single droplet vaporizes, for example, by an acoustic pulse, it may trigger mass transfer and bubble inflation from surrounding droplets. For in vivo imaging, such an event may cause unwanted bioeffects such as vessel occlusion. Even in the absence of bubble inflation within the microvasculature, circulating superheated nanodrops are expected to rapidly dissolve and expel their FC through the lung alveoli. The use of near-boiling or sub-boiling FCs, such as perfluoropentane (PFP, *n*-C_5_F_12_, bp = 29 °C) or perfluorohexane (*n*-C_6_F_14_, bp = 56 °C),^26^ circumvents this problem, but they also require much higher energies for vaporization. For example, acoustic droplet vaporization of PFP requires a mechanical index (MI) of 2.3 (e.g., 4 MPa peak negative pressure at 3 MHz), which exceeds the safety limit of 1.9 for medical ultrasound imaging and 0.8 for contrast-enhanced ultrasound imaging.^27^

To address this limitation, Shakya et al. designed vaporizable endoskeletal droplets.^1,28,29^ These droplets consist of a solid alkane hydrocarbon (HC) core encapsulated by a liquid PFP droplet and stabilized in an aqueous medium by a fluorosurfactant (H/F/W emulsion). Upon heating the droplet to near the melting temperature of the HC core, interfacial mixing between the FC and HC lowers the spinodal of the FC, leading to vaporization of the droplet at a temperature below the boiling point for pure PFP. The lowering of the spinodal temperature is a product of the poor miscibility between the PFP and linear alkane hydrocarbons, captured by the exchange parameter (χ). The critical temperature (*T*_c_) of the PFP depends on the exchange parameter through the following equation, as derived by Shakya et al.,

1 $$T_{c}=\frac{1.1443\times \Delta H_{V}T_{r}}{\Delta H_{v}+RT_{r}\left(P_{o}x_{F}\exp\left(\chi {\left(1-x_{F}\right)}^{2}\right)\right)}+69.898$$

where Δ*H*_v_ is the enthalpy of vaporization for PFP, *T*_r_ is room temperature (298 K), *R* is the universal gas constant, *P*_o_ is the vapor pressure of PFP, and *x*_F_ is the mole fraction of PFP at the diffuse interface between FC and HC. At low values of *x*_F_, *T*_c_ is reduced, as is the spinodal temperature (*T*_s_ = 0.8*T*_c_ to 0.9*T*_c_), where vaporization occurs (Figure S1). Therefore, as the HC melts and interfacial mixing occurs, *T*_s_ drops to below the bulk temperature of the droplet, causing vaporization. This allows for significantly lower vaporization temperatures compared to pure PFP droplets, which vaporize between 65 and 105 °C (80–90% of critical temperature).

This configuration allows for the use of a more colloidally stable FC droplet with a low vaporization energy by tuning the melting temperature of the HC (i.e., choosing an HC with a melting temperature near 37 °C such as C_20_H_42_, C_21_H_44_, or C_22_H_46_). For odd-numbered alkanes, vaporization occurs near the rotator phase transition.^29^ Furthermore, the vaporization behavior can be altered by heating the droplets to above the melting temperature of HC (while in the sealed vial to avoid premature vaporization), followed by rapid quenching (rapid cooling through the crystallization temperature of HC) in an ice bath. Such heat treatment resulted in an increase in the vaporization temperature and the percentage of droplets that vaporized.^29^

Shakya et al. also demonstrated that the droplet morphology could be inverted by replacing the fluorosurfactant with phospholipid,^29^ following a report by Zarzar et al., who showed reconfigurable complex emulsions through the choice of HC vs FC surfactant.^30^ When using a fluorosurfactant (Krytox), the FC/water interfacial energy is reduced, resulting in H/F/W droplets (Figure 1a,c). Conversely, when a HC surfactant (phospholipid) is used, the HC/water interfacial energy is reduced, leading to F/H/W droplets (Figure 1b,d). The structures of the fluorosurfactant (Krytox) and lipid (DBPC) used in our study are shown in Figure S2.

**Figure 1:**
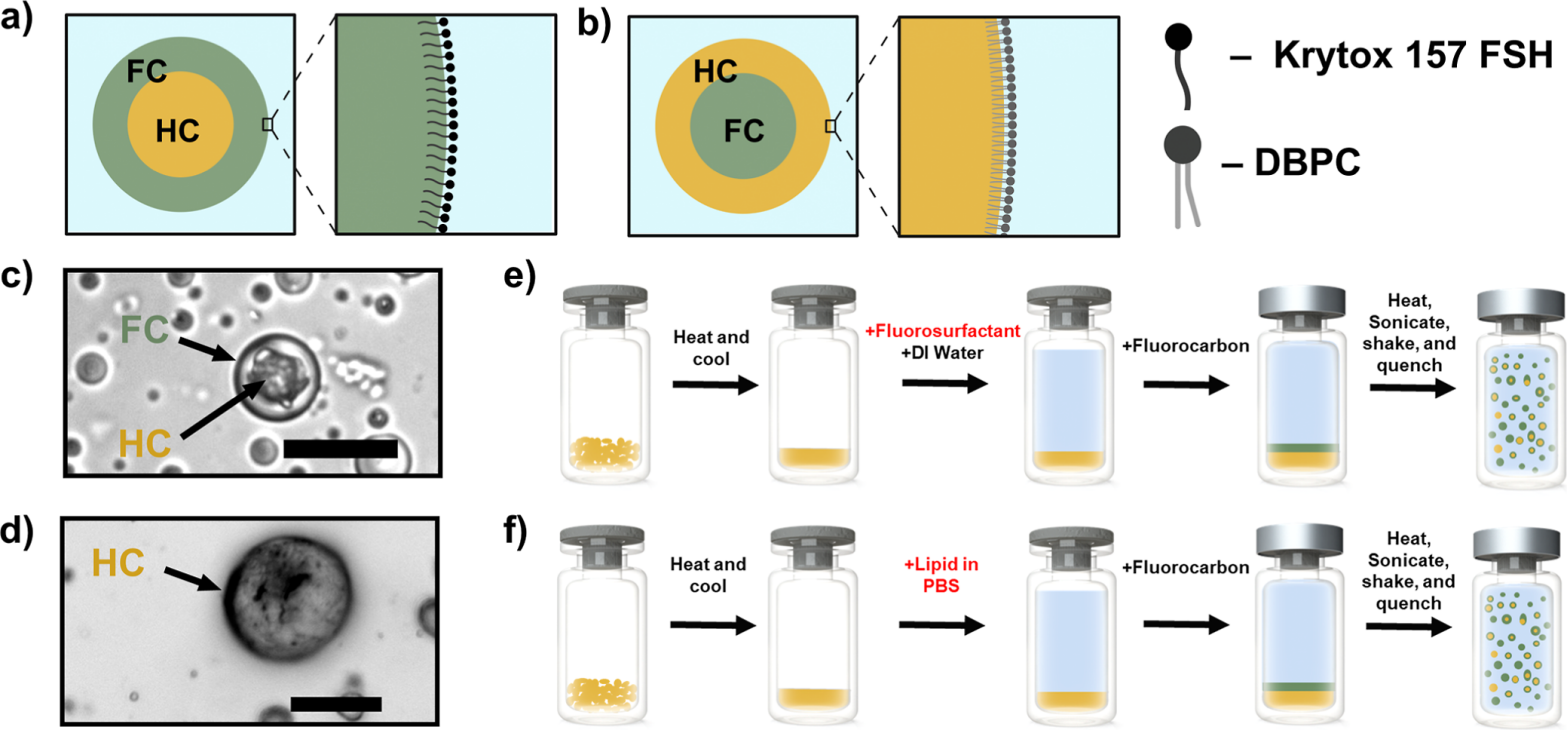
Design and synthesis of H/F/W endoskeletal and F/H/W exoskeletal droplets. (a) Diagram of an endoskeletal droplet with Krytox 157 FSH at the FC-water interface. (b) Diagram of an exoskeletal droplet with DBPC lipid at the HC-water interface. (c) Micrograph of an endoskeletal droplet. (d) Micrograph of an exoskeletal droplet. (e,f) Synthesis process steps involved for endoskeletal droplets (e) or exoskeletal droplets (f). Scale bars: 20 μm.

Monodisperse populations of both endoskeletal and exoskeletal droplets have been generated via microfluidics.^29^ However, droplet production via microfluidics faces challenges with low production rate, poor scalability and difficulty producing small droplets (nanodrops) suitable for intravenous injections. As such, there is motivation to investigate vaporizable droplets developed from a scalable one-pot synthesis approach and postproduction processing to refine their size distribution and vaporization behavior. Additionally, it is desirable to choose phospholipid over fluorosurfactant for improved biocompatibility. In this paper, we develop the one-pot synthesis and explore processing methods to control morphology, size distribution and vaporization behavior of lipid-coated F/H/W exoskeletal droplets. As a model formulation, we chose to focus on droplets made of *n*-eicosane (C20, *T*_m_ = 36 °C), due to the previously reported high vaporization efficiency compared to *n*-heneicosane (C21, *T*_m_ = 40 °C).^29^ These droplets were observed to vaporize below the physiological temperature (37 °C). Future work on HC mixtures may allow tuning of the vaporization temperature to occur at 37 °C with activation by ultrasound, photons or other energy sources. We demonstrate that, while the vaporization temperature is consistent between H/F/W endoskeletal and F/H/W exoskeletal droplets, the latter show a qualitatively different vaporization process with multiple nucleation events evolving more slowly due to confinement of the FC phase by the HC shell.

## Methods

### Materials

The following chemicals were used as received: *n*-perfluoropentane (PFP, *n*-C_5_F_12,_ 99%, FluoroMed, Round Rock, TX, USA), heretofore referred to as FC (FC); 1,2-dibehenoyl-*sn*-glycero-3-phosphocholine (DBPC) (>99%, Avanti Polar Lipids, Alabaster, AL, USA); 1,2-disteroly *sn*-gylcero-3-phosphoethanolamine-*N*-[methoxy­(polyethylene glycol)-5000] (DSPE-PEG5K) (>99%, Avanti Polar Lipids, Alabaster, AL, USA); *n*-eicosane (*n*-C_20_H_42_, 99%, Beantown Chemical, Hudson, NH, USA), heretofore referred to as HC; Krytox 157 FSH oil (Miller-Stepenson Chemical, Danbury, CT, USA); chloroform (99.8%, Fisher Scientific, Waltham, MA, USA); and phosphate buffered saline (PBS) (Molecular Biologicals International, Irvine, CA, USA), filtered.

### Synthesis of H/F/W Endoskeletal Droplets

Endoskeletal droplets were produced by adapting a previously described shaking technique (Figure 1e).^28^ To produce endoskeletal droplets, 157 mg of *n*-eicosane (*n*-C_20_H_42_, HC) was weighed in a 3 mL glass serum vial. The solid HC was melted using a heated water bath set to 45 °C for 5 min and then recrystallized by quenching in an ice bath. Once recrystallized, 56 mg of Krytox was added to the vial along with 2.5 mL of ultrapure Milli-Q water, chilled at 4 °C. Next, 200 μL of chilled (−20 °C) liquid PFP (*n*-C_5_F_12_) was pipetted into the vial. The vial was immediately sealed with a rubber septum and an aluminum crimp cap. The sealed vial was returned to a heated water bath for 5 min to allow for the solid HC film to completely melt. The vial was then placed in a bath sonicator heated to 45 °C, sonicated at high power for 60 s, and then immediately transferred to a mechanical shaker for 45 s. After shaking, the droplets were quenched by swirling in an ice bath for 30 s and then allowed to rest in the ice bath for an additional 5 min.

### Synthesis of F/H/W Exoskeletal Droplets

A lipid film of DBPC and DSPE-PEG5K (9:1) was prepared, as previously described:^28^ 116 mg of DBPC and 84 mg of DSPE-PEG5K were dissolved in two separate vials of approximately 2–3 mL of chloroform. The contents of the vials were combined and mixed, and then, the chloroform was evaporated by flowing nitrogen over the headspace and then placing it under vacuum overnight to remove any residual solvent. The dried film was hydrated using 20 mL of PBS (final lipid concentration 10 mg/mL) by heating to 5 °C above the main phase transition temperature of the lipid (75 °C) for 40 min and then cooling to room temperature. The hydrated suspension was then sonicated by using a probe sonicator at low power (3/10) for 10 min to convert large multilamellar vesicles to small unilamellar vesicles. The lipid suspension was then chilled and stored at 4 °C.

Exoskeletal droplets were produced using a shaking technique, such as that of the endoskeletal droplets (Figure 1f). To produce the droplets, 157 mg of *n*-C_20_H_42_ was weighed in a 3 mL glass serum vial. The solid HC was melted using a heated water bath set to 45 °C for 5 min, then recrystallized by quenching in an ice bath. Once recrystallized, 2.5 mL of the lipid suspension was added to the vial. Next, 200 μL of chilled (−20 °C) liquid *n*-C_5_F_12_ was pipetted into the vial. The vial was immediately sealed with a rubber septum and aluminum crimp cap. The sealed vial was returned to the heated water bath for 5 min to allow for the solid HC film to completely melt. The vial was then placed in a bath sonicator heated to 45 °C, sonicated for 60 s, and then immediately transferred to a mechanical shaker for 45 s. After shaking, the droplets were quenched by swirling in an ice bath for 30 s, then allowed to rest in the ice bath for an additional 5 min. To refine the suspensions, bulk exoskeletal droplets produced by shaking were gravity filtered using a 20 μm nylon cell strainer (Pluriselect) placed on an orbital shaker (Figure 2a). As a control to compare vaporization thresholds, pure PFP droplets were fabricated by using the same protocol, except the HC was omitted from the formulation.

**Figure 2:**
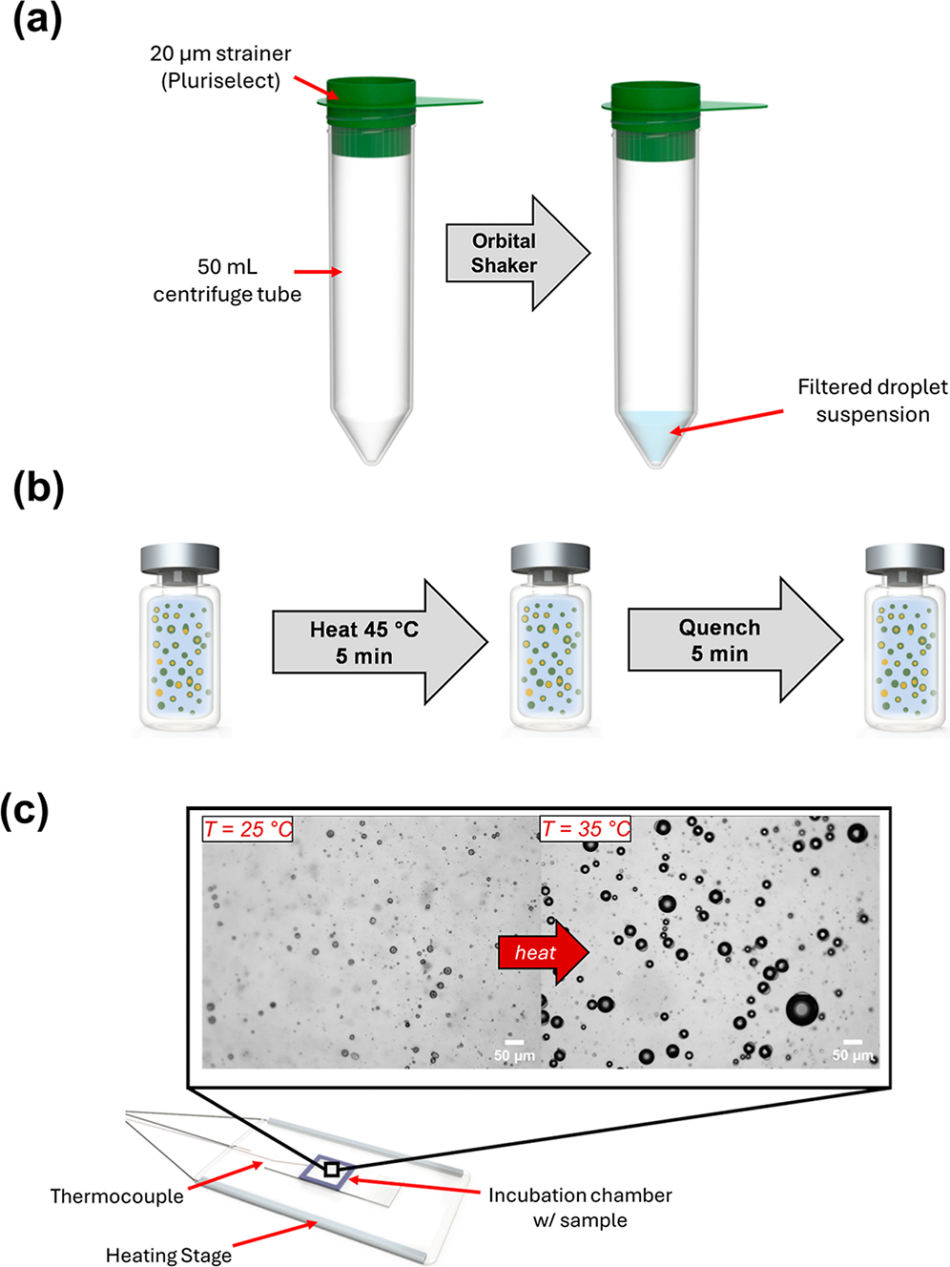
(a) Process for filtration of the exoskeletal droplets. (b) Approach to heat treating the droplets. The quenching conditions were altered to adjust the cooling rate of the droplets. (c) Set-up for thermal vaporization analysis of the droplets on the microscope stage.

### Heat Treatment of Droplets

The process for heat treating droplets was adapted from previous protocols^29^ and is shown in Figure 2b. The vials were heated to 45 °C for 5 min, followed by quenching under varying conditions to control the rate of cooling. Cooling methods included (1) swirling in an aqueous ice bath (∼1–2 °C) for 1 min and then allowing the sample to rest in the bath for an additional 4 min; (2) swirling in ethanol chilled to −20 °C for 15 s and then transferring to an ice bath for 4 min 45 s; and (3) quenching in a bath of canola oil, cooled to ∼4 °C for 5 min. The difference in bath temperature and thermal diffusivity of the liquid resulted in variations in the rate of cooling through the melting temperature of the HC, as shown in Figure S3. The impact of the cooling rate was assessed under the microscope, as described in the following section. To directly observe the effects of heat treatment on droplet morphology, a sample of the droplets under the microscope were heated to 45 °C, cooled to ∼5 °C, then heated again to 45 °C using a system of Peltier devices powered by a DC power supply and aluminum cooling blocks circulating chilled (∼4 °C) water (Figure S4). The aluminum blocks acted as a heat sink for the Peltier devices during the thermoelectric cooling of the microscope slide.

### Thermal Vaporization Experiments

The setup for thermal vaporization experiments is shown in Figure 2c. Nucleation and vaporization were observed using an Olympus BX51 bright-field microscope (Olympus, Waltham, MA). The sample was positioned under the microscope on a clear microscope heating stage, controlled by a PID controller (HWPT-384S Trans Well Plate Heater and mTCII microTemperature Controller, Cell MicroControls, Norfolk, VA, USA). The target temperature of the stage was set to 45 °C, and the sample was heated from room temperature to the target temperature in an exponential-rise fashion. In the case of pure PFP droplets, the sample was heated to 85 °C using two Peltier devices attached to a DC power supply (Agilent E3640A, Santa Clara, CA, USA). The temperature of the sample was recorded with a separate thermocouple. Videos of the droplets during thermal vaporization experiments were taken at a frame rate of 5 fps using a QImaging QIClick CCD Camera (Redwood City, CA). To determine the vaporization temperature of the sample, the temperature at which bubbles nucleated was marked using ImageJ, and the normalized number of bubbles formed as a function of temperature was fitted to a cumulative normal distribution curve in Prism 10 (GraphPad, Boston, MA). Experiments were performed on both filtered and unfiltered exoskeletal droplets. Impact of cooling rates on nucleation was assessed by dividing the concentration of gas nuclei observed in the microscope ROI by the concentration of droplets (as measured by the Multisizer, see Particle Sizing section). The concentration was adjusted to remove the impact of droplets that were FC only by multiplying by the fraction of droplets with an exoskeletal droplet morphology.

To demonstrate clinical utility, contrast enhancement of the filtered exoskeletal droplets was assessed using a clinical ultrasound scanner (Siemens Sequoia C512, Issaquah, WA, USA). The droplets were suspended in a carbomer gel (0.5% Carbopol 2050 ETD, Lubrizol, Wickliffe, OH, USA) to fix the bubbles in place following vaporization.^7^ After the polymer solid was dissolved in DI water, the droplets were added to the solution under stirring to a final concentration of 10^7^ droplets/mL. The pH was then adjusted to 7 using NaOH to form the gel and fix the droplets. The gel was then centrifuged at 500 RCF for 20 min to remove trapped air bubbles. Following centrifugation, the gel was loaded into a dialysis tube and fixed in a water bath. The water bath was then heated from room temperature to 45 °C using a sous vide while B-mode ultrasound imaging was performed at a MI of 0.1 and frequency of 8 MHz. The change in video intensity was measured in Fiji, normalized, and fit to a cumulative normal distribution curve to determine the vaporization temperature.

### Particle Sizing

Immediately after synthesis, the droplets were sized using a Multisizer 3 Coulter Counter (Beckman Coulter) equipped with a 30 μm aperture tube (range 0.6–24 μm). The Multisizer uses an electrozone sensing method to determine particle sizes and the concentration of a sample. For each measurement, 5 μL of droplets were diluted in 10 mL of Isoton II diluent (Beckman Coulter). Using the Multisizer, number and volume weighted size distributions were determined as well as the concentration of each droplet sample.

Optical microscopy was used to measure the size distributions of bubbles generated from endo- and exoskeletal droplets. Following thermal vaporization, 10 representative micrographs of bubbles from three separate samples and three independently made vials (9 total samples and 90 total images) were recorded at 4× magnification. This magnification was chosen to increase the upper diameter of detection to ∼300 μm, but limited the lower diameter limit to ∼15 μm. Using a custom MATLAB script, the images were first binarized to isolate the bubbles from their background, and then the “imfindcircles” function from the MATLAB Image Processing Toolbox was used to identify and measure the diameter of the isolated bubbles. To limit false positives and negatives, each analyzed image was visually inspected and the sensitivity of the function was adjusted as needed. Number- and volume-weighted size distributions were generated from the measurements for both endo- and exoskeletal droplets. A similar process was carried out to measure the size distributions of exoskeletal droplets prior to and following filtration; imaging of the droplets (before vaporization) was recorded at 10× magnification to size smaller droplets (Figure S5). Bubble concentrations were measured by dividing the total number of bubbles in the field of view by the volume of the fluid in the same field of view. To determine the vaporization efficiency, the number of bubbles per volume of sample was divided by the concentration of particles measured by the Multisizer and then adjusted to account for fraction of FC only droplets.

### Statistical Analysis

All statistical analyses were carried out in Prism 10 (Graphpad, Boston, MA). Two-sample, two-tailed unpaired *t*-tests were used to compare the means between data sets. One-way analysis of variables (ANOVA) was used to measure the relationships between cooling rate on nucleation and vaporization temperature. All measurements were performed in triplicate across three independent vials (total of nine measurements). An alpha value of 0.05 was used to determine the significance for all tests.

## Results and Discussion

### Droplet Morphology

Representative images of individual droplet morphologies are shown in Figure 3. Compared to endoskeletal and exoskeletal droplets formed via microfluidics,^5,29^ the stochastic nature of the shaking approach led to a variety of unique structures with varying ratios of HC and FC in each droplet. Similar structures were also obtained following an alternative sample preparation and shaking technique (Figures S8 and S9). Figure 3a shows typical structures found in H/F/W endoskeletal morphology, where fluorosurfactant stabilized the FC-water interface. As a result, numerous FC-only droplets were observed (93 ± 3% by number). Of the endoskeletal droplets, the relative amount of HC content varied between droplets and formed different structures, including spherical solids (Figure 3a­(i,ii), 1.6 ± 0.5%) and nonspherical solids (Figure 3a­(iii), 4.5 ± 1.7%). Emulsified water droplets in the FC phase were sometimes observed (Figure 3a­(iv), 1.0 ± 0.6%). Similar water inner phases have been observed in perfluorocarbon droplets using Krytox and its derivatives.^18^ The inner water phase allows for the loading of hydrophilic drugs and can lower the threshold for acoustic droplet vaporization.^18,31^

**Figure 3:**
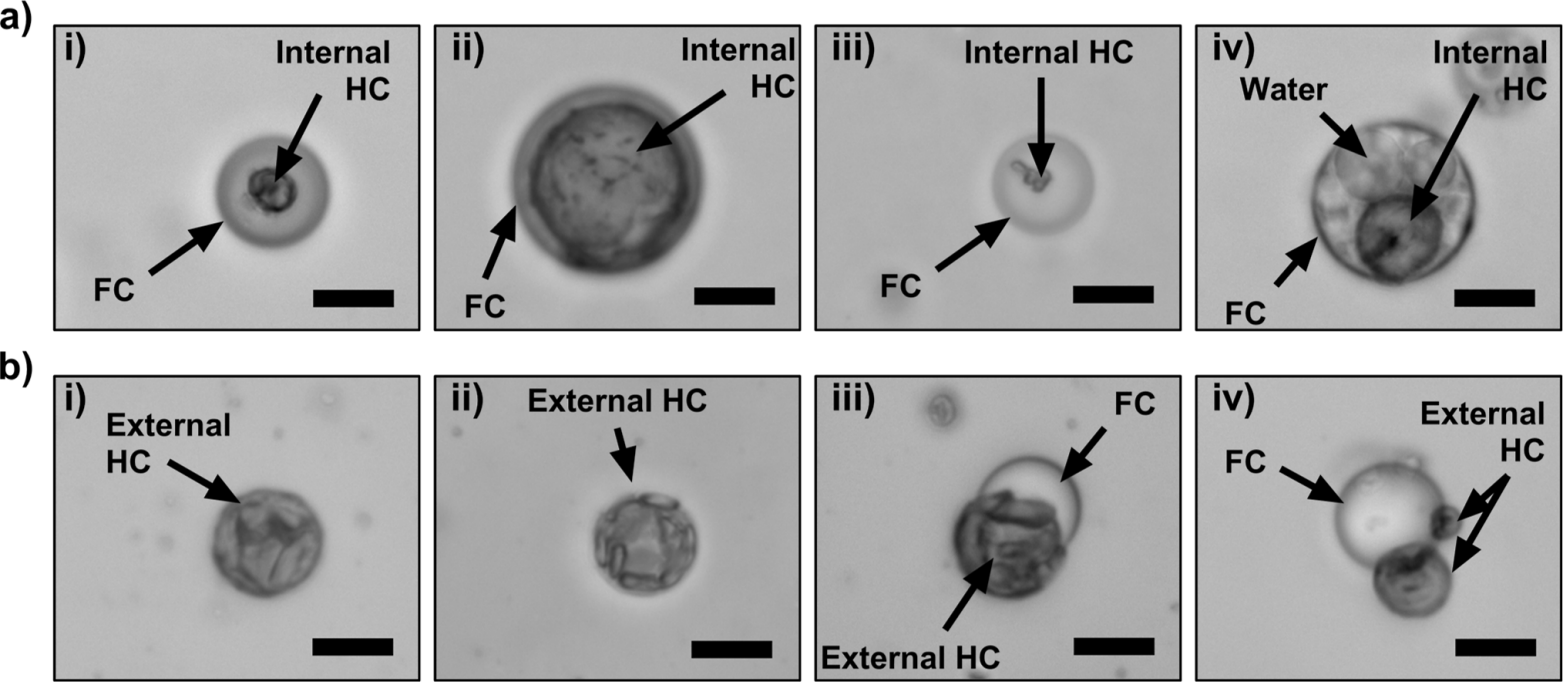
Brightfield microscopy images showing morphology of (A) H/F/W endoskeletal and (B) F/H/W exoskeletal droplets. Endoskeletal droplets had a smooth spherical structure with a liquid FC outer layer entrapping solid HC as its core. Exoskeletal droplets had a solid HC outer shell structure (often crumpled) with FC entrapped as the core or partially covered with a HC shell. Scale bar: 10 μm.

Figure 3b shows representative F/H/W exoskeletal droplet structures, formed through lipid stabilization of the HC-water interface. Like the endoskeletal droplets, the relative ratio of HC to FC varied between droplets produced by our one-pot synthesis method, and different morphologies and HC shell thicknesses were observed. Future work could explore microfluidic techniques to produce uniform droplets with controlled HC:FC ratios and monodisperse size and structure. However, at the present time, microfluidic methods have not been developed to produce F/H/W exoskeletal droplets with sufficiently small diameter (<10 μm). The observed morphologies were reproducible and seen across multiple batches and included complete encapsulation of the FC droplet with crumpled solid HC surface structure (Figure 3b­(i,ii)), partially encapsuled FC by the HC exoskeleton forming a Janus-type structure (Figure 3b­(iii)), and multiple Janus structure^32^ (i.e., multiple HC structures held together by a partially exposed FC droplet) (Figure 3b­(iv)). Of the exoskeletal droplets, the complete encapsulation morphology (Figure 3b­(i,ii)) was most commonly observed (14 ± 4%), appearing on the top of the microscope slide (buoyant, HC-rich region). The partially encapsulated and Janus-like particles (Figure 3b­(iii,iv)) were much less common (5 ± 1%) and found on the bottom of the slide with the sedimentary, FC-rich droplets but were still reproducible and observed across different droplet batches. The partial encapsulation of FC was also noted by Shakya et al.,^29^ who noted Janus-like structures that formed from microfluidics-produced exoskeletal droplets when the HC flow rate was insufficiently high. The remaining fraction of droplets consisted of FC-only droplets (81 ± 4%).

The choice of surfactant not only controls the morphological structure of the droplet but also can impact the biocompatibility. Unlike Krytox, which is a lubricant and not approved for any pharmacological use, the phospholipid shell used for the exoskeletal droplets is similar to that used for clinically approved microbubble ultrasound contrast agents^33^ and research-grade injectable nanodrop ultrasound contrast agents,^34^ and its biocompatibility is well characterized. As such, there is motivation to utilize lipid-coated exoskeletal droplets over Krytox-coated endoskeletal droplet formulations for future in vivo applications.

### Observation of Vaporization Behavior

A representative timelapse of the vaporization of a H/F/W endoskeletal droplet is shown in Figure 4 where the contrast has been increased using Fiji. As the droplet is heated to its vaporization temperature, a sudden expansion of the FC phase was observed (Figure 4f), triggered by melting of the HC core. The transition from droplet to bubble was rapid, on the order of milliseconds. In the frames prior to this rapid vaporization of the FC phase (Figure 4b–e), a vapor embryo was observed as a dark spot formed near the HC/FC interface, indicated by the yellow outline. The vapor embryo grew and spread at the HC/FC interface, supporting the hypothesis that vaporization is triggered by surface melting and interfacial mixing.^28^ An annotated video of the timelapse shown in Figure 4 is included in Supplemental Video S1.

**Figure 4:**
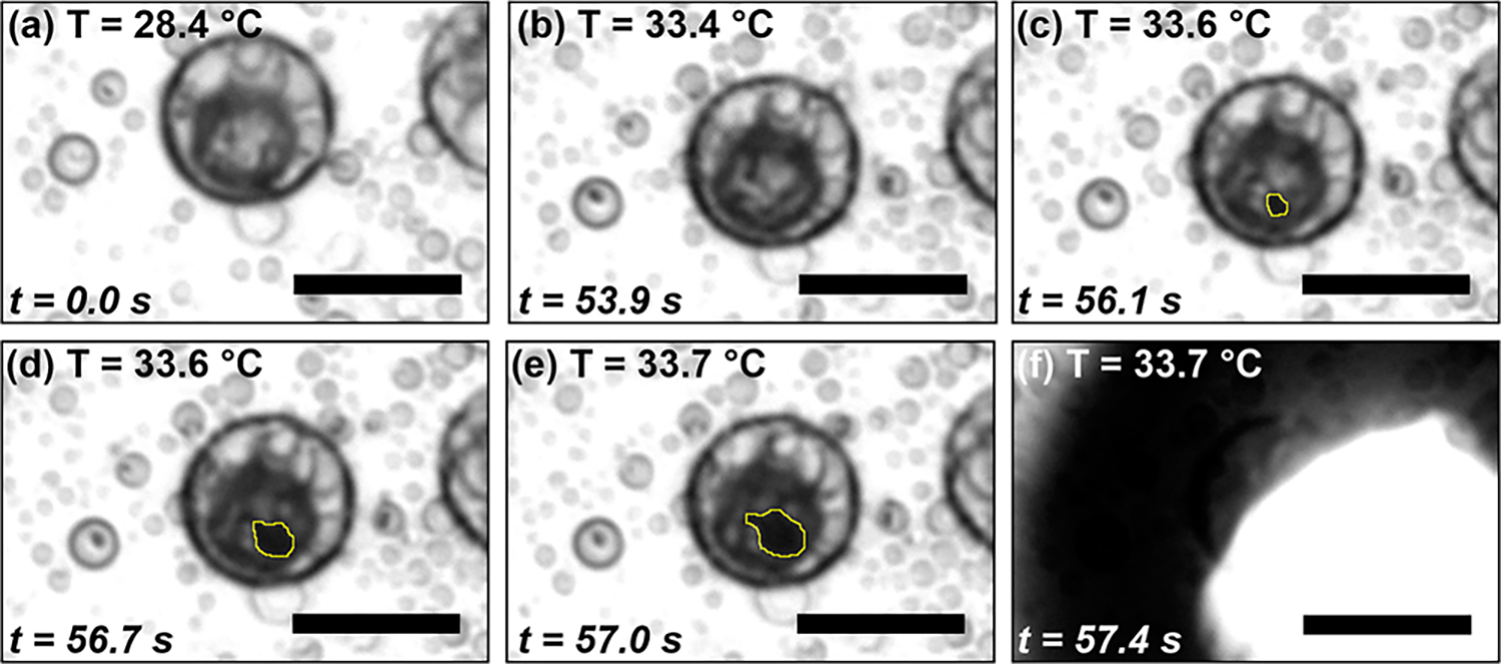
Timelapse of endoskeletal droplet vaporization. (a) Micrograph of endoskeletal droplet well below the HC melting temperature. (b,c) Upon heating, a vapor embryo, seen as a small dark spot, appears on the HC core, indicated by the yellow outline (found via image thresholding in Fiji). (d,e) The vapor embryo expands at the HC/FC interface. (f) The droplet suddenly vaporizes into a bubble. See Supplemental Movie S1 for video of this timelapse. Scale bar is 20 μm.

Additional videos of nucleation and vaporization of endoskeletal droplets can be found in the Supporting Information (Videos S2, S3 and S4). In Videos S2, S3 and S4, a similar darkening of the HC core can be seen immediately prior to vaporization.

A representative timelapse of an F/H/W exoskeletal droplet is shown in Figure 5. Similar to the endoskeletal droplets, as the droplet approaches its vaporization temperature, the HC begins to melt and vaporization of the droplet occurs. However, the time course of exoskeletal vaporization is much longer, occurring over several seconds. As the droplet was heated, vapor embryos were observed as dark spots indicated by the arrows (Figure 5b). Upon additional heating, multiple vapor nuclei emerged and expanded (Figure 5c,d). These nuclei eventually coalesced into a single bubble (Figure 5e), and the HC exoskeleton melted and formed a liquid-lens-like structure on the surface of the bubble (Figure 5f). A video of the timelapse shown in Figure 5 can be found in Supplemental Video S5, where formation and growth of the multiple vapor nuclei can be readily visualized.

**Figure 5:**
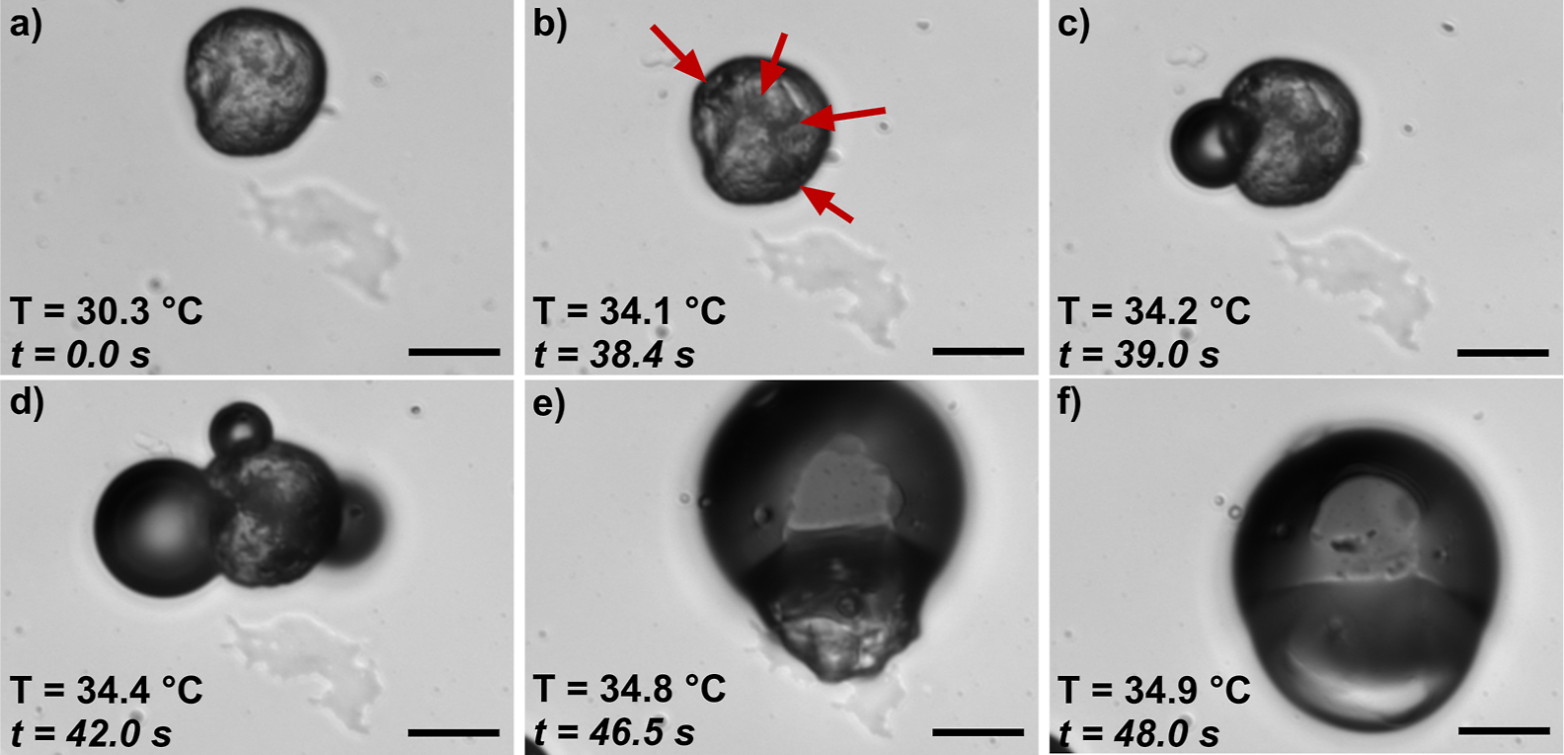
Timelapse of exoskeletal droplet vaporization. (a) Exoskeletal droplet prior to heating. (b) Early on, vapor embryos are observed as dark spots, indicated by the red arrows. (c) A bubble emerges and (d) expands, while additional bubbles form on the surface of the HC. (e) These bubbles coalesce and (f) the HC melts, forming a lens structure on the surface of the bubble. A video of this timelapse can be found in Supplemental Video S5. Scale bar is 20 μm.

Additional videos of exoskeletal droplets vaporizing can be found in Supplemental Videos S6, S7 and S8. In Video S6, an exoskeletal droplet that has completely encapsulated the FC phase is shown. As the droplet was heated, a dark spot formed on the right side of the droplet that emerged as a vapor nucleus. Multiple nuclei formed and coalesced into a singular bubble, and the HC melted into the lens-like structure, similar to what is shown in Figure 5e,f. When the exoskeletal droplet morphology exhibited partial encapsulation of the FC (Videos S7 and S8), the darkening within the HC phase was still observed. However, as the gas nucleated and contacted the liquid FC, the nucleus rapidly expanded into a large gas bubble, similar to vaporized endoskeletal droplets. In the case of Video S8, the bubble coalesced with a neighboring bubble. For instances of complete encapsulation, the solid HC exoskeleton acted as a rigid barrier against expansion of the gas, causing a much slower overall vaporization process; this resistance to expansion was not present in endoskeletal or partially encapsulated Janus-type droplets.

The observation of the dark spot formation is consistent with vapor embryo formation and expansion in pure perfluorocarbon droplets, seen in high-frame rate video microscopy studies.^27,35–37^ The time between the formation of the gas embryo within the liquid and the vaporization of the pure FC droplets was on the order of nanoseconds. However, the process of vaporization was observed to be much slower for our exoskeletal droplets. Our qualitative observations suggested that liquid FC trapped within solid HC vaporized first, causing small gas nuclei to form. The solid HC exoskeleton constricted growth of the gas nuclei until the solid sufficiently melted for the nuclei to emerge into the surrounding water and eventually coalesce (as observed in Figure 5). The rate of vaporization is limited by the mechanical resistance of the HC solid shell, prolonging the process compared with pure FC drops.

### Size Distributions and Thermal Vaporization Curves for the Raw Droplets

Size distributions are listed in Figure 6a,b. The number-weighted mean (±standard deviation) diameter of the endoskeletal droplets was 1.30 ± 0.05 μm (median of 1.12 ± 0.03 μm) and the volume-weighted mean diameter was 4.85 ± 1.97 μm (median of 4.89 ± 1.54 μm), showing high polydispersity as produced by the shaking method. For the exoskeletal droplets, the number-weighted and volume-weighted mean diameters were 1.19 ± 0.05 μm (median 0.96 ± 0.02 μm) and 8.44 ± 2.42 μm (median 7.05 ± 3.66 μm), respectively. The mean diameters between endoskeletal and exoskeletal droplets were significantly different (*p* < 0.01 for both number and volume weighting). The concentration of exoskeletal droplets (1.38 ± 0.43 × 10^9^ particles per mL) was significantly greater than that for the endoskeletal droplets (0.14 ± 0.06 × 10^9^ particles per mL) (*p* < 0.001).

**Figure 6:**
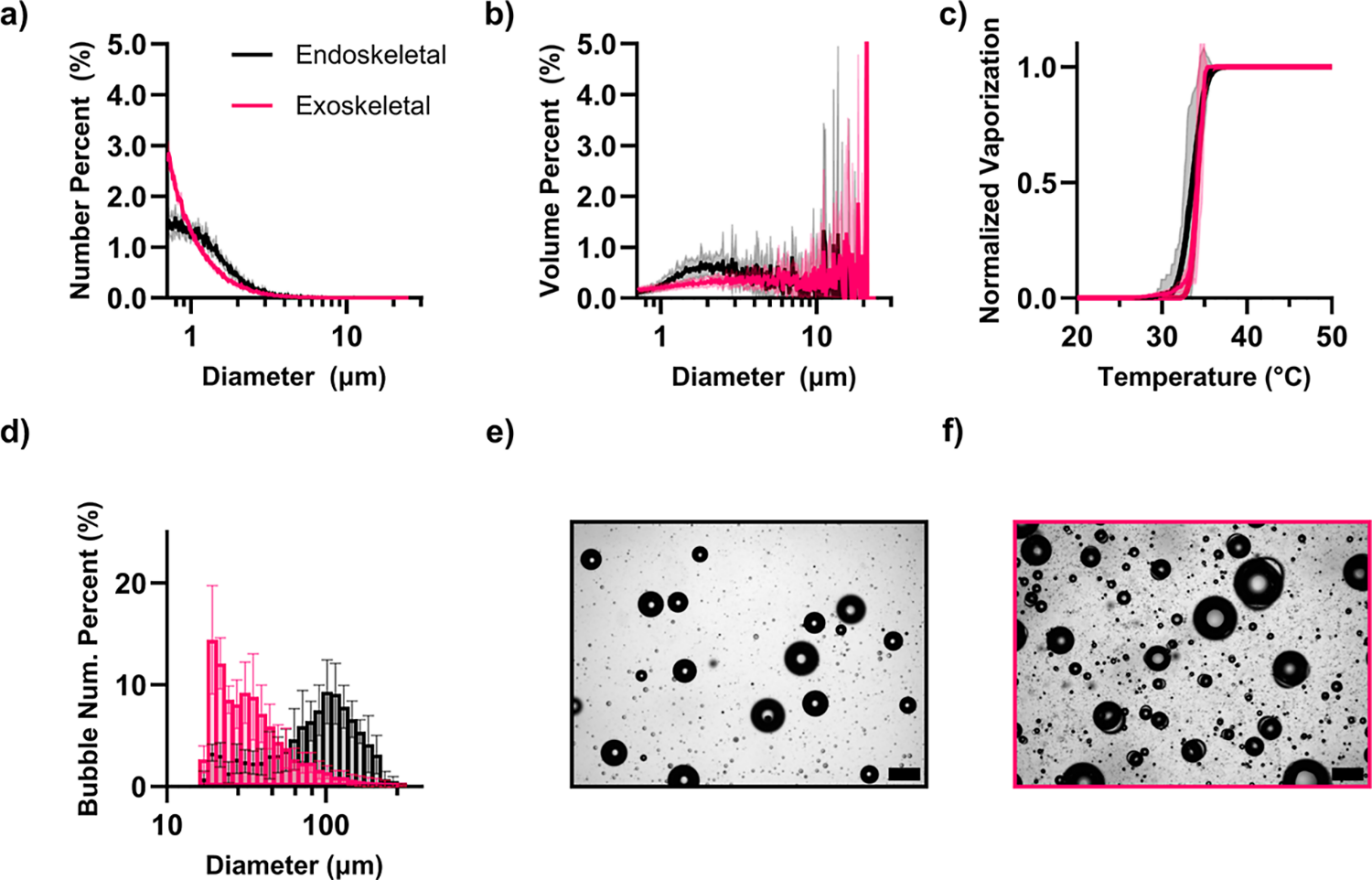
(a) Number-weighted and (b) volume weighted size distributions of endoskeletal (black) and exoskeletal (pink) droplets, determined from Multisizer measurements. Mean diameter (number and volume weighted) for exoskeletal droplets was found to be significantly smaller compared to endoskeletal droplets (*p* < 0.01). (c) Thermal vaporization results for both droplet types. No significant difference in the vaporization temperature was found. (d) Number-weighted optical size distribution of bubbles following vaporization. (e) Endoskeletal droplets produced mean bubble sizes larger than the exoskeletal droplets (f). Scale bar is 200 μm in (e,f).

Thermal vaporization curves for the endoskeletal and exoskeletal droplets are shown in Figure 6c. Based on the fitted curve, the thermal vaporization (±standard deviation) was fitted to 33.6 ± 1.2 °C for endoskeletal droplets and 34.2 ± 0.7 °C for exoskeletal droplets, and they were not statistically different (*p* = 0.22). As both use the same HC and FC species, this was expected; neither size, concentration, morphology (exoskeletal vs endoskeletal) nor choice of surfactant appeared to affect the vaporization temperature. Both droplet morphologies vaporized at temperatures much lower than pure PFP droplets (*p* < 0.0001), which vaporized at 65.0 ± 12.9 °C (Figure S10). This threshold for vaporization corresponds to the expected spinodal temperature of PFP.

Size distributions and representative images of the bubbles generated from the endoskeletal and exoskeletal droplets are shown in Figure 6d–f. The mean diameter of bubbles vaporized from exoskeletal droplets (41.2 ± 5.3 μm) was significantly smaller than for endoskeletal droplets (95.7 ± 6.3 μm) (*p* < 0.0001). Exoskeletal droplets produced a higher concentration of small bubbles. This suggests that, despite having an equal amount of FC and HC used in their fabrication, exoskeletal droplets favor a more HC-rich droplet (and less FC), leading to the production of smaller bubbles. We next investigated filtration of larger exoskeletal droplets.

### Filtration of Exoskeletal Droplets

The results of filtering the exoskeletal droplets are listed in Figure 7. Prior to filtration, the droplet number-weighted mean diameter (determined via optical microscopy) was measured to be 17.7 ± 2.9 μm; the droplet volume-weighted mean diameter was 52.6 ± 15.6 μm. Following vaporization, the bubble mean diameters were 40.5 ± 5.7 and 179.2 ± 39.6 μm (volume weighted) (Figure 7a,b). Representative images of the resulting bubbles are shown in Figure 7c.

**Figure 7:**
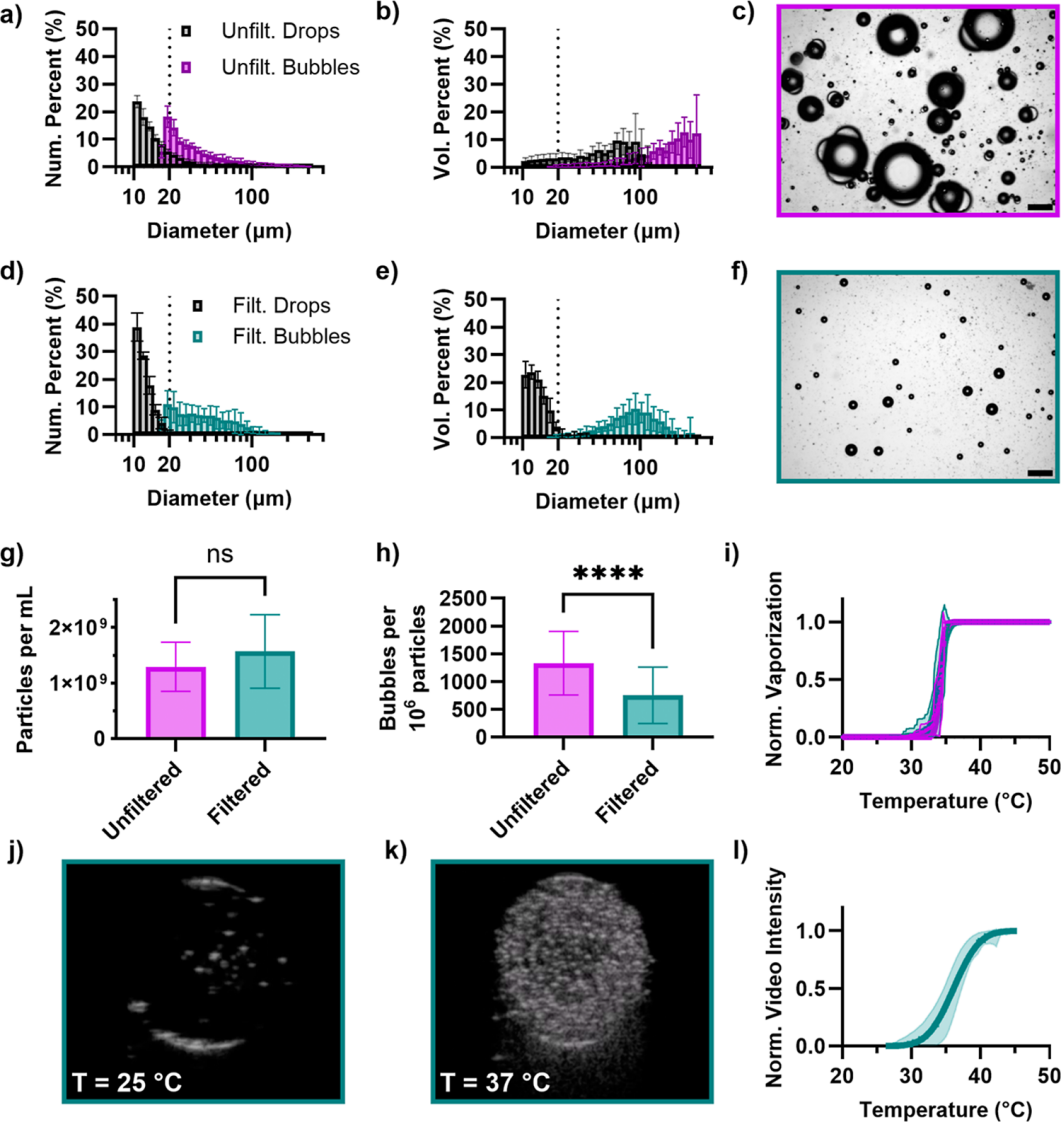
(a) Number-weighted and (b) volume-weighted size distributions of raw (unfiltered) exoskeletal droplets and resulting bubbles. (c) A representative micrograph of the bubbles formed following thermal vaporization. Scale bar is 200 μm. (d) Number-weighted and (e) volume-weighted size distributions of exoskeletal droplets and resulting bubbles following filtration with a 20 μm cell strainer. (f) A representative micrograph of the bubbles formed following thermal vaporization, highlighting the removal of bubbles larger than 100 μm. Scale bar is 200 μm. (g) Particle concentration of exoskeletal droplets before (raw) and following filtration, measured by the Multisizer. (h) Concentration of bubbles produced following thermal vaporization, normalized by particle concentration. (i) Thermal vaporization curves for the raw and filtered droplets. No significant change in vaporization temperature was observed. (j) B-mode ultrasound images (MI = 0.1, 8 MHz frequency) below and (k) above the thermal vaporization temperature of the filtered exoskeletal droplets. (l) Normalized video intensity of the thermal vaporization assessed under ultrasound. Mean vaporization temperature was not significantly different than the value measured using microscopy.

Following filtration, the measured droplet diameters were 12.3 ± 0.26 μm (number weighted) and 13.7 ± 0.58 μm (volume weighted), which was significantly smaller than the raw droplets (*p* < 0.00001 and *p* < 0.0001, respectively). The resulting bubble sizes were 42.9 ± 11.0 μm (number weighted) and 94.6 ± 19 μm (volume weighted) (Figure 7d,e). The bubble volume-weighted mean diameter was significantly lower than that of the bubbles produced from the raw droplets (*p* < 0.0001); there was no significant change in number-weighted diameter (*p* = 0.52). While these mean values excluded the smaller droplets within the sample, the significant reduction in mean diameter of the large droplet population indicates successful removal of large droplets and reduction of bubble size by filtering.

Given the large number of small droplets in the raw samples, the concentration did not change significantly due to filtration (Figure 7g). The droplet concentration, droplet morphology, and size distribution were also stable over the course of 3 weeks when stored at 4 °C (Figure S11). However, the fraction of droplets that vaporized (number of bubbles per volume divided by initial number of droplets per volume), significantly decreased (*p* < 0.0001, Figure 7h). The overall vaporization efficiency of both droplet types (raw and filtered) is much lower than what was previously measured for monodisperse droplets,^29^ which can be attributed to the presence of nearly pure FC or HC droplets in the polydisperse samples. Additional processing to remove these populations is expected to increase the efficiency of vaporization. No significant change in vaporization temperature was observed (34.2 ± 0.6 °C prefiltration and 34.0 ± 1.0 °C postfiltration, *p* = 0.55, Figure 7i). This result is consistent with our observation above that the vaporization temperature is independent of droplet size and morphology.

To show clinical utility, the contrast enhancement below and above the vaporization temperature of the filtered droplets is shown in Figure 7j,k. Below the vaporization temperature, very little contrast enhancement is observed in the gel. Above the vaporization temperature, a strong contrast is observed. Using the ultrasound setup, the vaporization temperature of the droplets was measured to be 36.1 ± 2.9 °C (Figure 7l). This was not statistically different from the value measured from microscopy experiments (*p* = 0.34).

### More Rapid Cooling Increased the Number of Nuclei for Exoskeletal Droplets

Figure 8 shows the results of rapid cooling during the heat treatment of exoskeletal droplets. For unfiltered exoskeletal droplets, one-way ANOVA showed a significant increase (*p* < 0.0001) in the number of nucleation sites for increasing cooling rate (Figure 8a,b). A similar result was obtained for filtered droplets (*p* < 0.001, Figure 8d,e).

**Figure 8:**
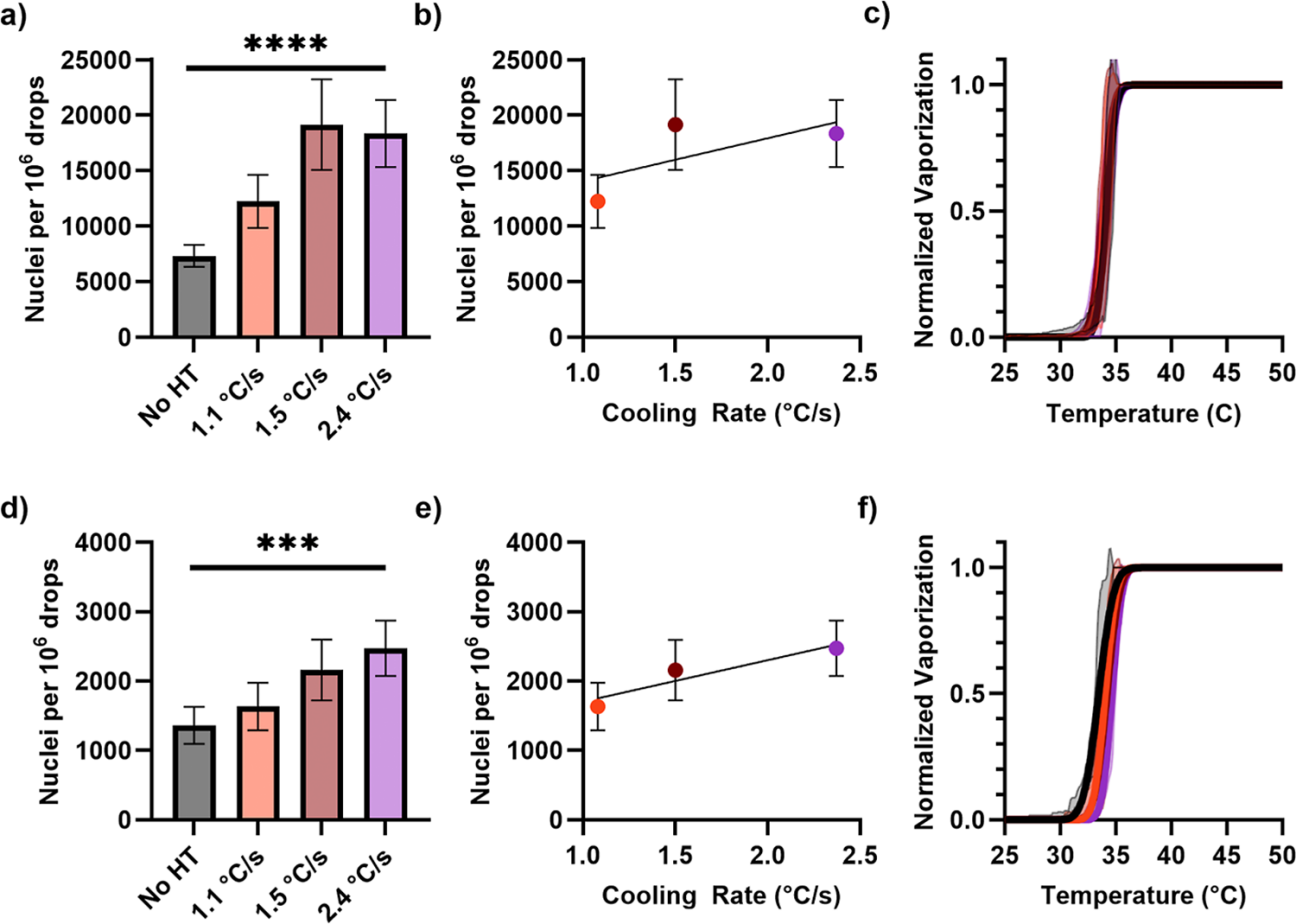
(a) Effect of cooling rate on the number of gas nuclei formed during thermal vaporization, normalized by particle concentration. Data is for raw (unfiltered) exoskeletal droplets. (b) As the cooling rate through the melting temperature of the HC increased, the number of nuclei formed increased. (c) No change in vaporization temperature was seen. (d) Effect of cooling rate on nucleation for filtered exoskeletal droplets. (e) For the number of nucleation sites, a similar relationship seen for filtered droplets as (b) unfiltered. (f) Effect of cooling rate on vaporization temperature. A monotonic relationship was present, but differences between groups were not significant.

Similar analysis indicated no significant change for raw or filtered droplets in the vaporization temperature due to the cooling rate (Figure 8c,f). This result is consistent with our results above that the droplet morphology did not affect the vaporization temperature. However, it is different than previous results by Shakya et al. with microfluidic endoskeletal droplets, where the vaporization temperature was shown to increase following heat treatment.^28^ One potential explanation could be the polydisperse nature of our droplets here, both in size and in morphology, leading to a wider range of vaporization temperatures for individual droplets.

To probe the mechanism for the increase in the number of nucleation sites, we directly observed droplets undergoing a heat treatment cycle (Figure 9). The droplet initially melted without vaporizing into a gas bubble. Upon cooling, the HC-rich droplet condensed a few degrees below its bulk melting temperature, and we observed fluid FC domains form on the surface of the HC (Figure 9c). These FC droplets coalesced and partitioned into crevices on the HC solid (Figure 9d). Upon further cooling, we observed darkening and contraction of the HC solid (Figure 9e); this darkening correlates to the rotator-to-crystalline phase transition, which can occur several degrees below the melting temperature of the HC.^38^ Upon heating the droplet again, we observed similar vaporization as shown in Figure 4: nucleation, expansion and coalescence of two bubbles and finally melting of the HC into a lens on the bubble surface (Figure 9g–l).

**Figure 9:**
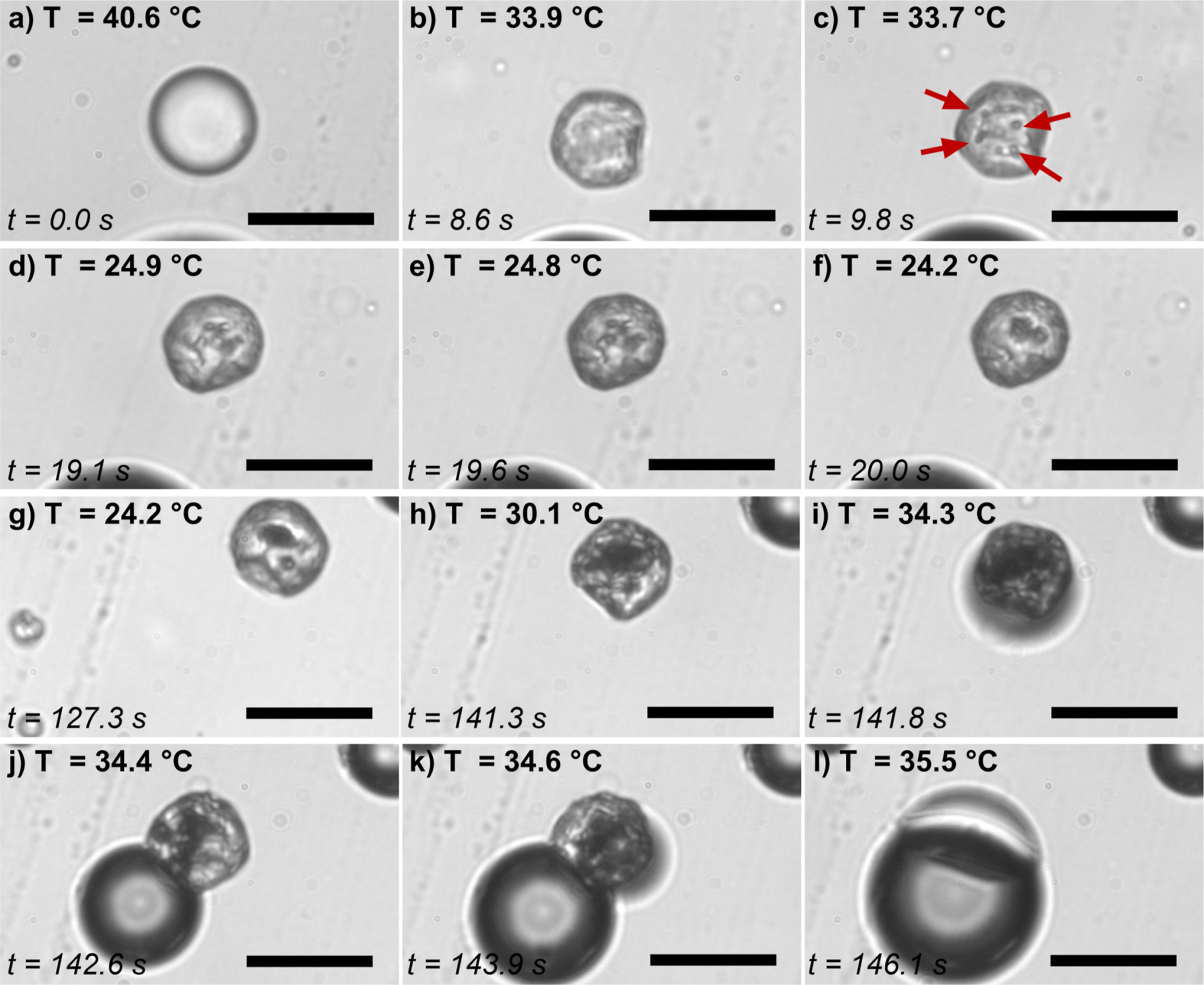
Heat treatment of an exoskeletal droplet. (a) Droplet was initially melted with no bubble attached. (b,c) Upon cooling, the FC phase separated from the solid HC and (d) partitioned into crevices of HC. (e,f) Upon further cooling, the HC underwent a rotator-to-crystalline phase transition, indicated by darkening and contraction of the solid. (g) The same droplet then underwent heating and (h–j) a bubble nucleated from a crevice on the solid HC. (k) A second bubble nucleated and coalesced before the HC completely melted (l) into a lens-structure on the surface of the bubble. Scale bar is 20 μm. See Video S9 for video of the timelapse.

During the typical heat treatment process, the droplets are placed in a sealed vial and heated above the melting point of the HC species. Heating in a sealed vial suppresses vaporization. Melting of the HC phase is supported by microscopy images showing a smooth, uniform and spherical appearance (Figure 9a). However, the FC does not appear to vaporize by this heating process, as bubbles are not observed in the vial macroscopically or on individual droplets under microscopy (Figure 9a). Therefore, both the HC and FC appear to be in the liquid phase and are miscible in the droplets after they are heated. Prior work estimated up to 1 mol % dissolved PFP could exist in an eicosane melt for our temperature range.^28^ Upon quenching, defined as the rapid cooling process whereby the prefabricated drops are cooled to ∼4 °C after they are heated, we observe the HC form a solid phase, observed by microscopy as rough, wrinkled and nonspherical in appearance (Figure 9b–f). Upon subsequent heating, the FC phase vaporizes, observed by microscopy as a highly contrasting bubble.

In our observation, quenching of the HC phase into its rotator phase appeared to induce phase separation of the FC from the HC, which then partitioned into crevices of the HC. Rapid cooling rates would favor solid nucleation over growth, increasing the number of crevices in the HC solid. The rapid cooling rate would also decrease the time for the FC drops to coalesce before the rotator-to-crystalline transition, increasing the number of HC crevices filled with FC. The rotator-to-crystalline phase transition is known to increase in density of the solid,^39^ leading to the formation of nanopores.^40,41^ Due to wetting of the FC liquid to solid HC (interfacial energy of 1–2 mN/m),^30,42^ the existing crevices and nanopores would favorably fill with the FC,^40,43,44^ potentially increasing the likelihood for vaporization due to a localized increase in FC-HC surface area.^29^ This phase separation and cooling phenomenon offers a potential mechanism for the increase in nucleation sites observed for exoskeletal droplets quenched at higher cooling rates.

## Conclusions

Here, we report on the formulation and characterization of novel vaporizable exoskeletal droplets formed by a one-pot synthesis. These exoskeletal droplets are more biocompatible than their endoskeletal counterparts due to the use of phospholipid, rather than fluorosurfactant, as the emulsifying agent as well as their smaller size. This biocompatibility increases their utility for applications such as contrast-enhanced ultrasound imaging,^12–14^ photoacoustic imaging^5^ and in vivo radiation detection.^11^ Additionally, the smaller size of the droplets and vaporized bubbles from filtered exoskeletal droplets renders them less likely to occlude microvessels in circulation, although we have not yet tested these droplets in vivo. Interestingly, exoskeletal droplets exhibit qualitatively different vaporization behavior, favoring multiple nucleation sites compared to their endoskeletal counterparts. The number of nucleation sites increased with heat treatment and quenching. Microscopy observations of heat-treated exoskeletal droplets suggested the presence of FC liquid pools contained in crevices of the HC solid shell, which served as nucleation sites. While the observed vaporization temperature of this system was below body temperature, future work will focus on alternative hydrocarbons (single phase or mixtures) and microstructures that produce vaporization slightly above 37 °C for biomedical imaging applications. Furthermore, due to the stochastic nature of droplet formation by the shaking method, the droplet compositions and structures within a given sample were heterogeneous. Future work will be aimed at better characterizing and controlling the exoskeletal droplet uniformity. Overall, these results highlight the tunability of exoskeletal droplet morphology, biocompatibility and vaporization behavior through surfactant selection during one-pot synthesis and postproduction processing.

## Supplementary Material





















## Notes

Plot of boiling, critical and spinodal temperature at the FC/HC interface; chemical structure of DBPC and Krytox; analysis to determine quenching rate; method for optical sizing of droplets and bubbles; micrographs of exoskeletal and endoskeletal droplets populations and number composition of exoskeletal and endoskeletal droplet morphology; alternative approach to producing droplets and resulting structures; thermal vaporization of pure PFP droplets; and stability of filtered exoskeletal droplets and exoskeletal droplet microstructures following quenching (PDF)
Vaporization of endoskeletal droplet presented in Figure 4 (MP4)
Vaporization of alternate endoskeletal droplet (MP4)
Vaporization of alternate endoskeletal droplet (MP4)
Vaporization of alternate endoskeletal droplet (MP4)
Vaporization of exoskeletal droplets presented in Figure 5 (MP4)
Vaporization of alternate exoskeletal droplet (MP4)
Vaporization of a partially encapsulated exoskeletal droplet (MP4)
Vaporization of alternate, partially encapsulated exoskeletal droplet (MP4)
Heat treatment of exoskeletal droplet (MP4)

## §

Institute of Biomedical Engineering, University of Oxford, Oxford, England

## Abbreviations

HC: hydrocarbon
FC: fluorocarbon
DBPC: 1,2-dibehenoyl-*sn*-glycero-3-phosphocholine
DSPE-PEG5K: *N*-(methylpolyoxyethyleneoxycarbonyl)-1,2distearoyl-*sn*-glycero-3-phosphoethanolamine
DI: deionized water
PBS: 10× phosphate buffered saline
