# Supplementary material for: Thermally Activated Vaporization of Fluorocarbon-In-Hydrocarbon Exoskeletal Droplets
Source: ACS Omega. 2026 May 6;11(19):27884–95. doi: 10.1021/acsomega.5c11095 (PMC13191537; doi:10.1021/acsomega.5c11095)
Supplement: Supplementary file 1 [file ao5c11095_si_001.pdf]

Supplementary information for

Thermally activated vaporization of fluorocarbon-in-hydrocarbon exoskeletal droplets

William N. Frantz,[1] Awaneesh Uphadyay,[2] Mark A. Borden[1,2*]

[1]Biomedical Engineering Program, University of Colorado, Boulder, CO 80309, United States.

[2]Paul M. Rady Department of Mechanical Engineering, University of Colorado, Boulder, CO 80309, USA

This PDF file includes:

Supplementary Figures S1 to S12
Captions for Supplementary Videos S1 to S9

Other Supplementary Materials for the manuscript include the following:

Supplementary Videos S1 to S9

Figures:

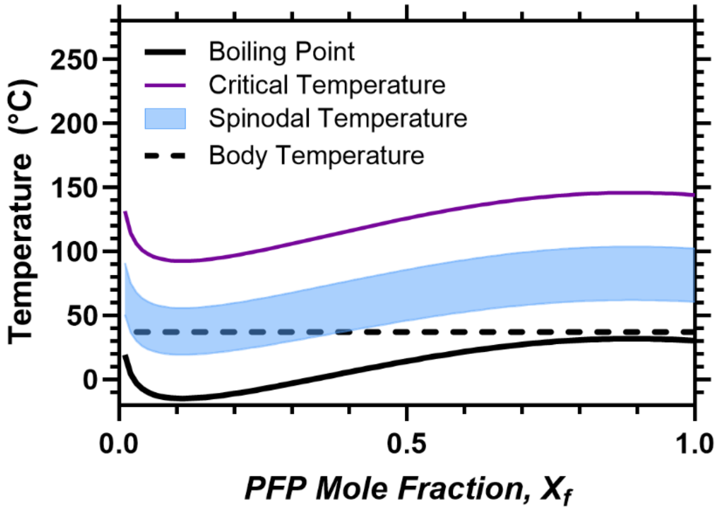

Figure S1. Boiling point, critical temperature and spinodal temperature (80-90% of T_c) depression at the HC/FC interface (Equation 1 in the main text). As the mole fraction of PFP (x_f) decreases, the spinodal temperature drops sufficiently low for vaporization to occur. Figure adapted from Shakya et al., 2020.

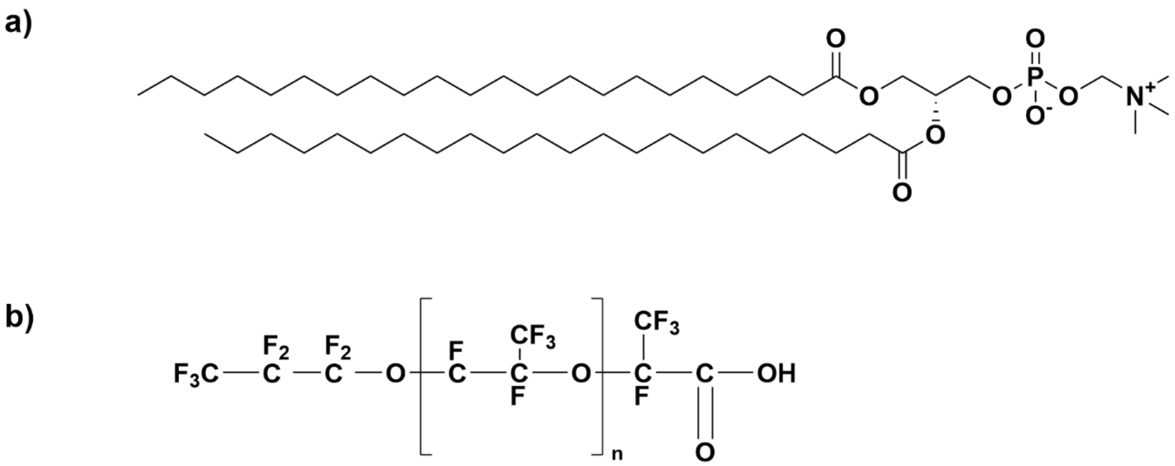

Figure S2. (a) Chemical structure of DBPC; (b) Chemical structure of Krytox 157 FSH, n = 41.

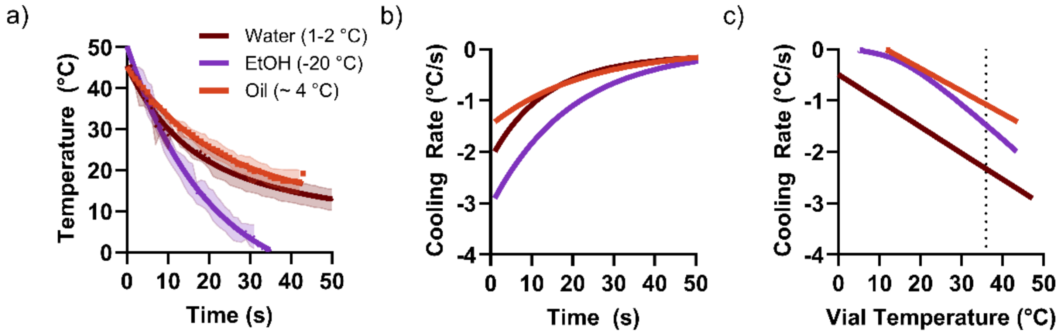

Figure S3. (a) Experimental cooling curves were obtained by quenching a sealed vial in different baths (see Methods). The data was fit with an exponential decay. (b) Cooling rates determined by the negative of the derivative of the cooling curves from part a). (c) The cooling rate through the melting temperature of the HC.

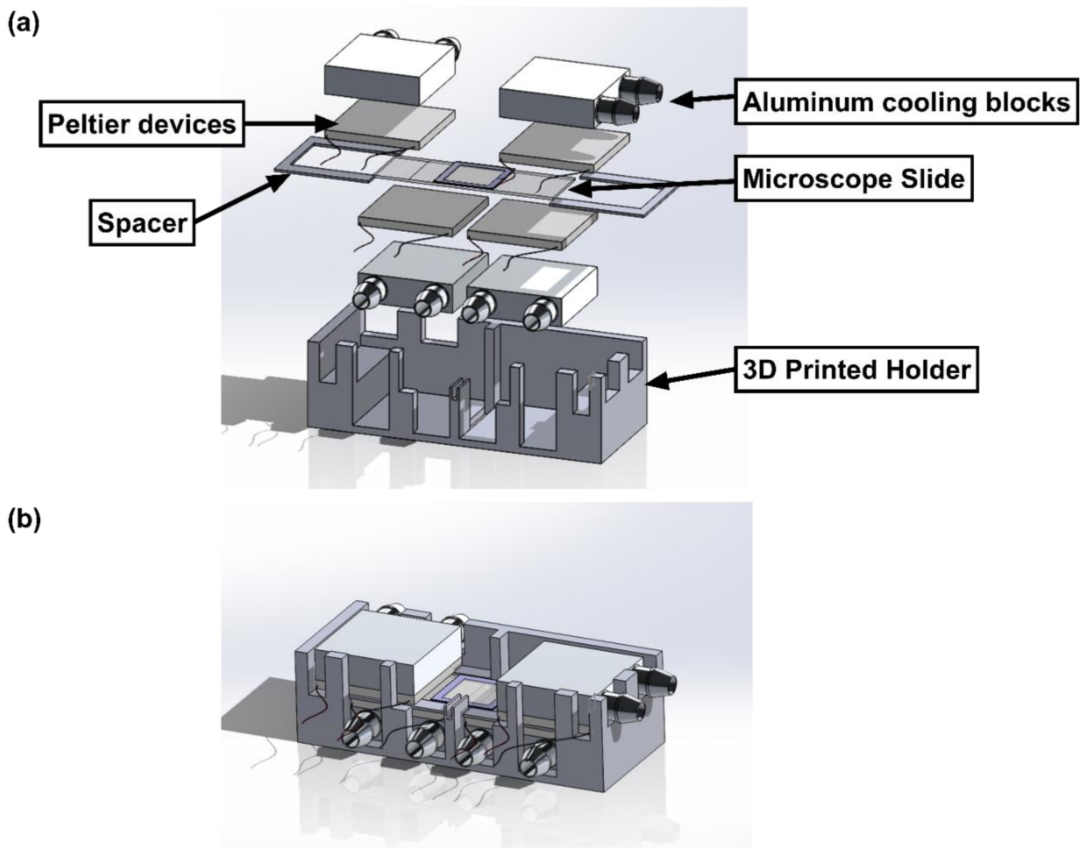

Figure S4. 3D model of cooling set-up for cooling stage in (a) exploded view (b) condensed view. Chilled water was circulated through the aluminum cooling blocks and acted as a heat sink for the Peltier devices during thermoelectric cooling.

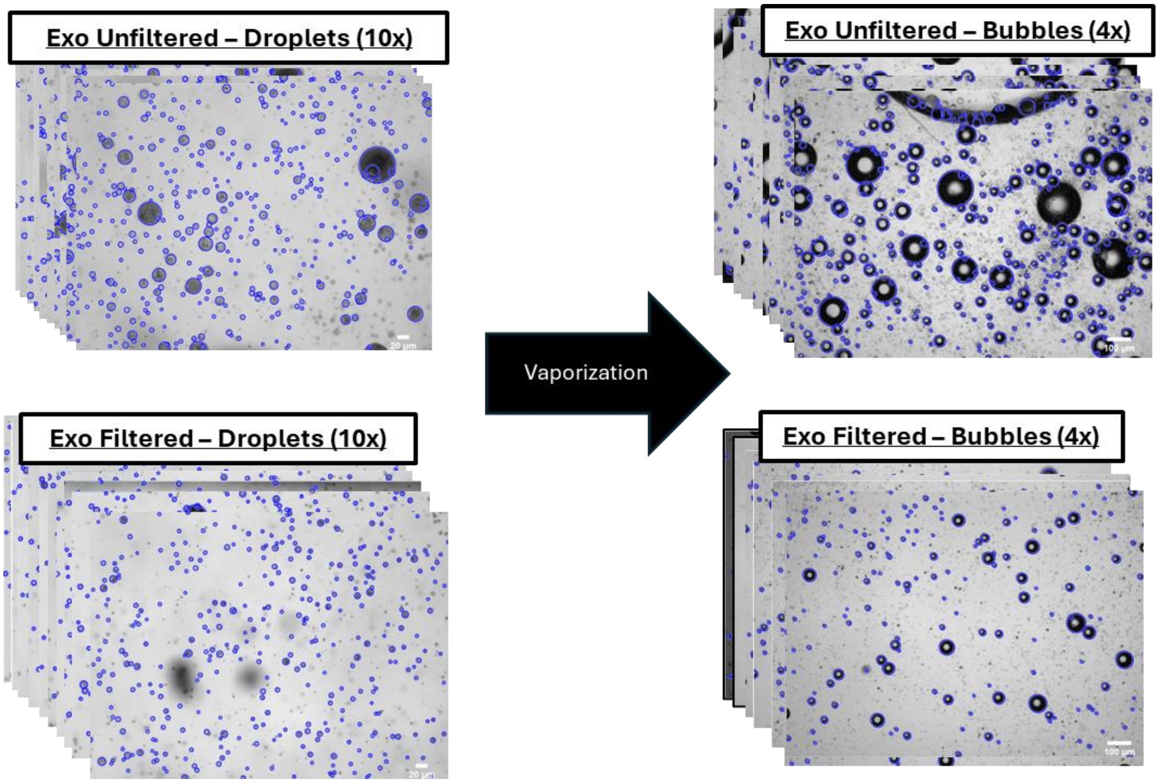

Figure S5. Optical sizing of the exoskeletal droplets and bubbles before filtration and after filtration. Visual inspection shows an obvious removal of the larger droplets and bubbles.

Determination of Morphology Prevalence

To determine the fraction of each morphology as shown in Figure 3, populations of endoskeletal (Figure S6a-b) and exoskeletal droplets (Figure S6c-d) were imaged on a hemocytometer. Both the bottom layer (infranatant) and top layer (supernatant) were imaged. Prior to imaging, the droplets were given five minutes to sediment (in the case of FC-rich drop) or rise to the coverslip. Using these micrographs, total droplets counts were determined using the MATLAB imfindcircles function. When counting the droplets, a lower diameter of 3 μm was used to omit the impact of droplets that remained in suspension and were not imaged. This cutoff was determined based on the Peclet number, where a value greater than 10 indicates the propensity for particles of this size to settle out of the suspension (Figure S7a, Equation S1).

$$Pe = \frac{8\pi g \Delta\rho a^4}{3kT} \quad \text{(S1)}$$

where g is the acceleration due to gravity, $\Delta\rho$ is the difference between the density of the droplet and the surrounding water, a is the radius of the particle, and kT is the thermal energy. The density of the droplet is a function of the relative amount of fluorocarbon volume fraction (ϕ_f) compared to the hydrocarbon. Note at $\phi_f \approx 0.26$ the droplets become neutrally buoyant and the Peclet number drops to zero. At volume fractions higher than this value, the droplets sink to the bottom of the slide; at volume fractions lower than 0.26, the droplets float to the cover slip.

After the total number of droplets within the size range were counted, the number of droplets of each morphology were counted manually in Fiji. From these counts, the fraction of each morphology was determined, as shown in Figure S7b-c. The measurements were performed in triplicate across three different vials (nine total measurements) for both endoskeletal and exoskeletal droplets.

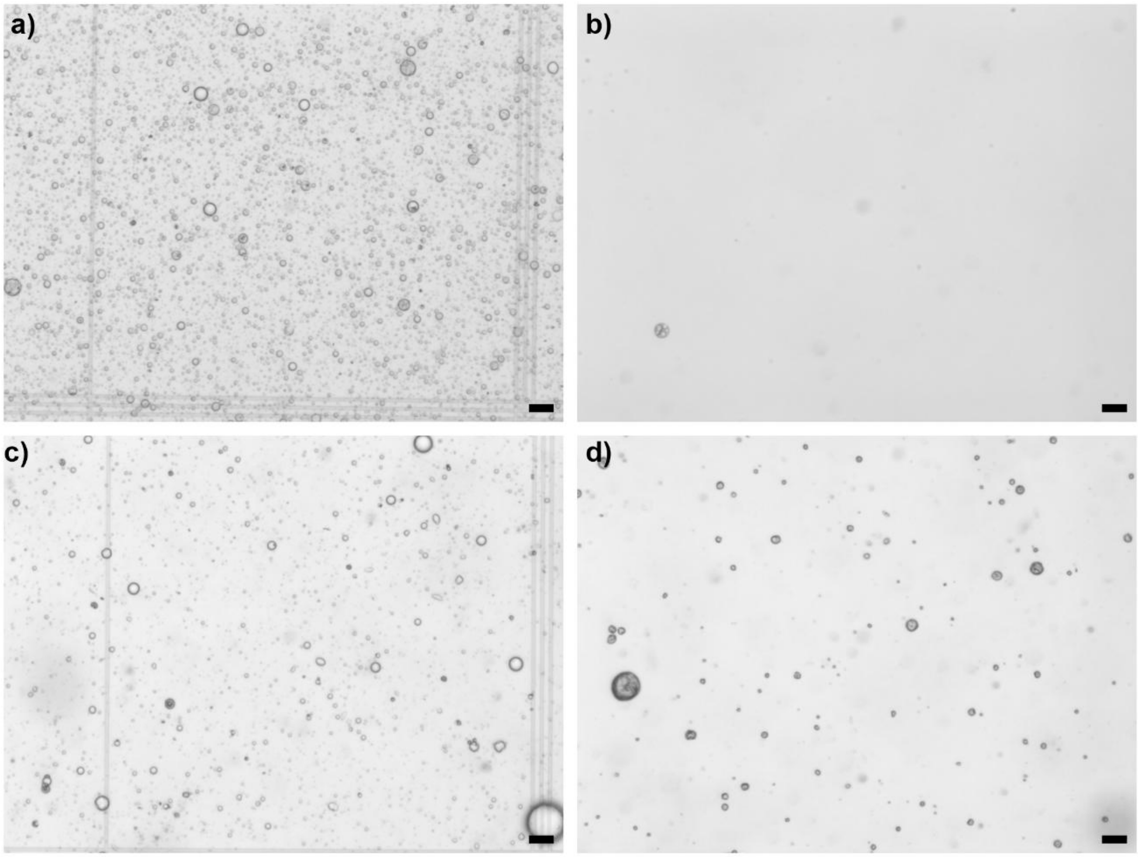

Figure S6. Representative images of a polydisperse population of endoskeletal droplets and exoskeletal droplets. (a) Example infranatant of endoskeletal droplet population, used to measured fraction of each morphology, present in Figure S7. (b) Supernatant of endoskeletal droplet sample. Few to no droplets were seen in the supernatant. (c) Infranatant of exoskeletal droplet population showing Janus-type droplets and (d) Supernatant of exoskeletal droplets showing the HC-encapsulated droplets. Scale bar: 20 µm.

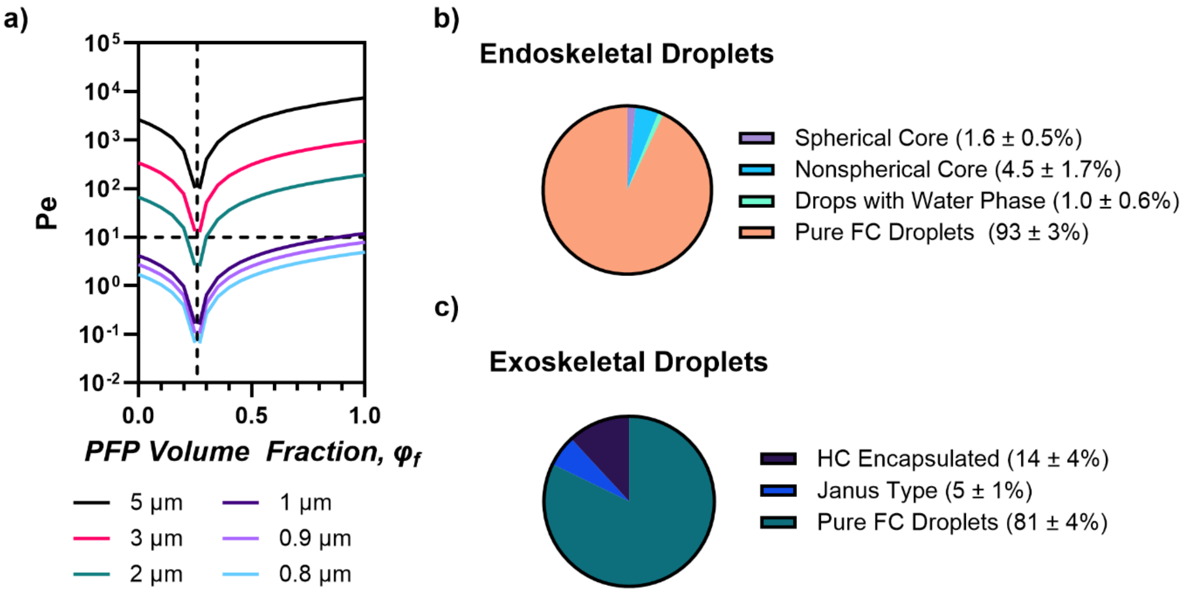

Figure S7. (a) Calculated Peclet numbers based on radius of the droplets and volume fraction of perfluorocarbon (PFP). Droplets with a Peclet number above 10 will sediment out of solution. Note when $\phi_f \approx 0.26$, the droplets become neutrally buoyant and remain suspended. (b) Number composition of endoskeletal droplet morphologies, as shown in Figure 3a. (c) Number composition of exoskeletal droplets morphologies, as shown in Figure 3b. Scale bar: 20 µm.

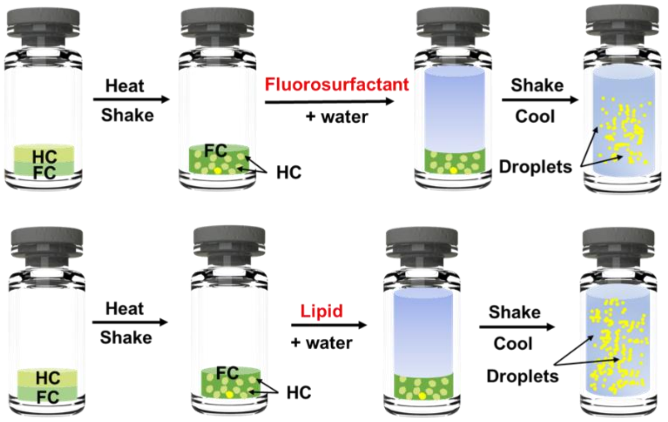

Figure S8. Alternative approach to producing polydisperse endoskeletal and exoskeletal droplets. HC and FC are first sealed, heated, and shaken together to mix the phases. Heated aqueous fluorosurfactant or lipid is added to the sealed vial using a syringe. The mixture is mechanically shaken and quenched to form droplets.

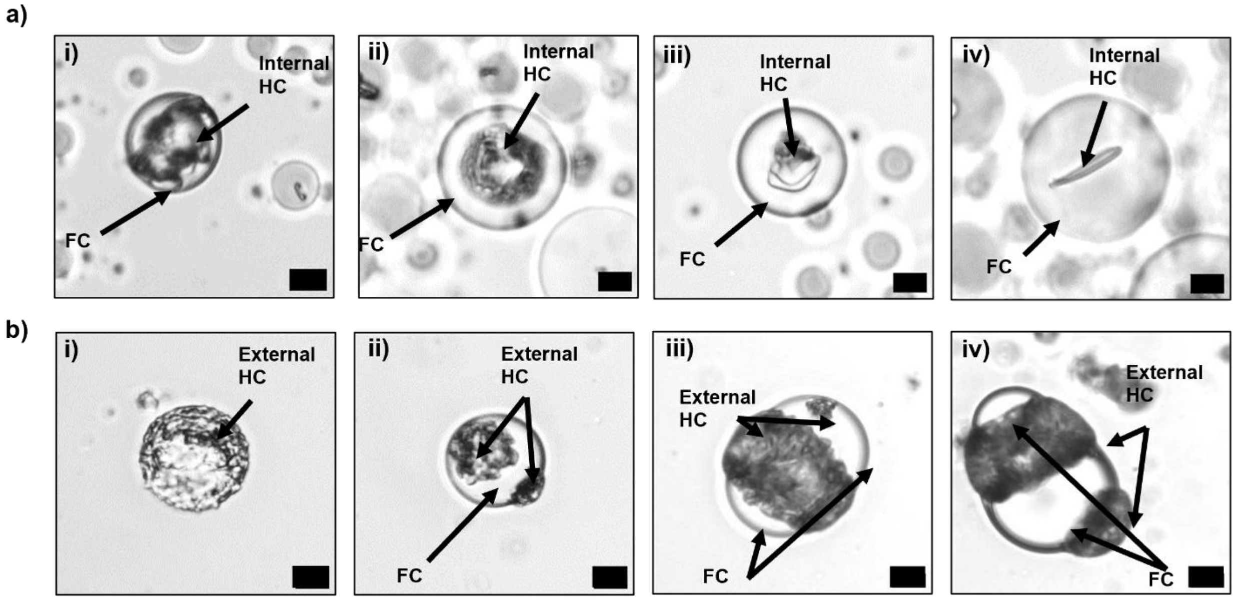

Figure S9. Brightfield microscope images of representative (a) endoskeletal droplets and (b) exoskeletal droplets produced with the method presented in Figure S5. Exoskeletal droplets show hydrocarbon outer shell structure with fluorocarbon entrapped as core or partially covered with hydrocarbon shell. Endoskeletal droplets have smooth spherical structure of fluorocarbon outer layer entrapping solid hydrocarbon as core. Scale bar 10 μm.

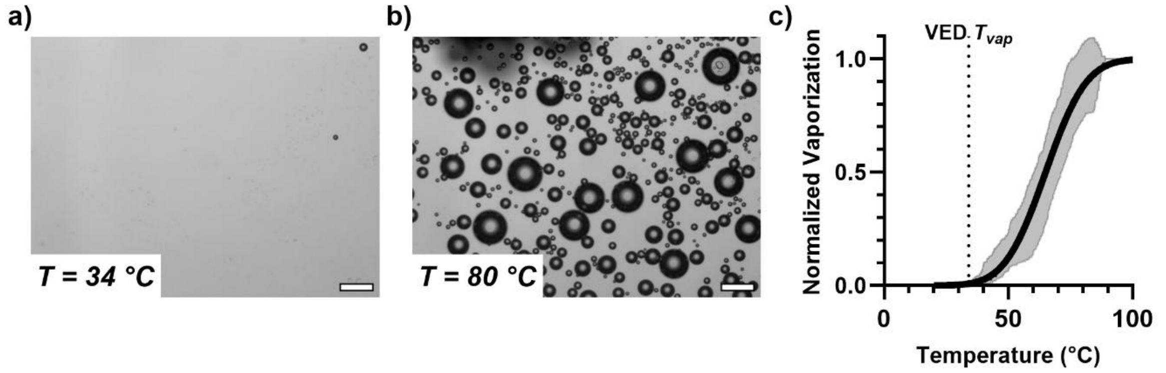

Figure S10: (a) Sample of pure PFP droplets at 34 °C (T_{vap} of vaporizable exoskeletal droplets (VEDs)), with minimal bubbles present in the sample. (b) Upon heating to 80 °C, pure PFP droplets are heated past their spinodal temperature and vaporize. Scale bar: 200 µm. (c) Thermal vaporization of pure perfluoropentane droplets, fitted to a cumulative normal distribution curve. Mean vaporization was 65.0 ± 12.9 °C.

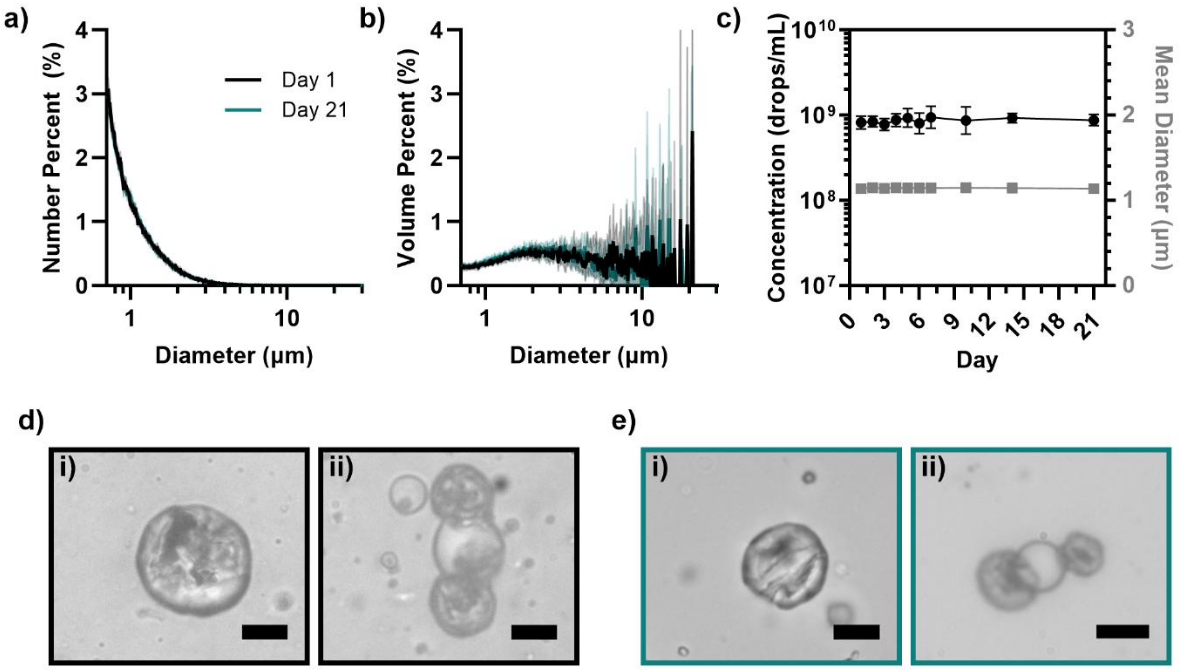

Figure S11: (a) Number-weighted and (b) volume-weighted size distribution of filtered exoskeletal droplets on the day they were made (Day 1) vs after two weeks storage at 4 °C (Day 14). (c) Measured concentration and mean diameter of the droplets over the course of the two weeks. (d) Exoskeletal droplets on day 1 showing typical (i) HC-encapsulated and (ii) Janus-type morphology, similar to structures in Figure 3. (e) These morphologies are still present in the samples after two weeks of storage. Scale bar: 10 μm.

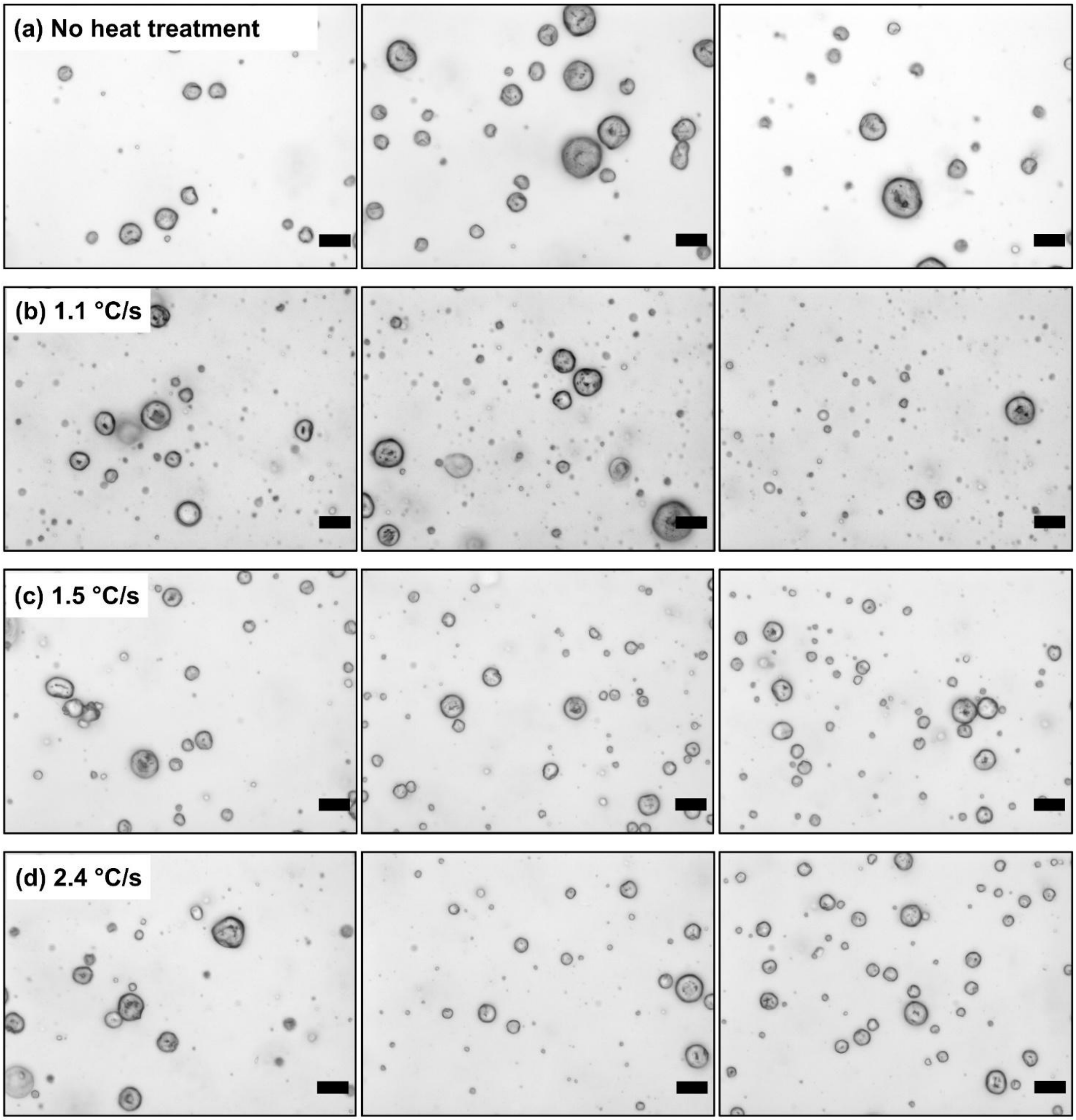

Figure S12. Micrographs of exoskeletal droplets at various cooling rates. (a) Micrographs of exoskeletal droplets prior to heat treatment. Following heating in a water bath, droplets were quenched in (b) chilled oil (1.1 °C/s), (c) chilled water (1.5 °C/s) or (d) ethanol (2.4 °C/s). No noticeable changes in structure of the HC phase were observed. Scale bar is 20 μm.

Supplementary Video Captions:

Video S1 Vaporization of endoskeletal droplet presented in Figure 4. The video is presented in regular speed followed by 0.5x speed. Arrow indicates the darkening of the HC core.

Video S2 Vaporization of alternate endoskeletal droplet. The video is presented in regular speed followed by 0.5x speed. An arrow indicates the darkening of the HC core.

Video S3 Vaporization of endoskeletal droplets with inner water phases. The video is presented in regular speed followed by 0.5x speed. An arrow indicates the darkening of the HC core.

Video S4 Vaporization of additional endoskeletal droplet. An arrow indicates the darkening of the HC core.

Video S5 Vaporization of exoskeletal droplets presented in Figure 5. The video is presented in regular speed. Nucleation is marked by red arrows.

Video S6 Vaporization of exoskeletal droplet, presented in regular speed. Formation of dark spot is marked with an arrow. Note the multiple bubbles that nucleate from the bubble.

Video S7 Vaporization of a partially encapsulated exoskeletal droplet. Video is shown in regular speed followed by 0.25x speed. Red arrows mark the formation of a dark spot. The spot expands and merges with the liquid droplet, causing sudden expansion into a bubble.

Video S8 Vaporization of a partially encapsulated exoskeletal droplet. Video is shown in regular speed followed by 0.25x speed. Red arrows mark the formation of a dark spot. The spot expands and merges with the liquid droplet, causing sudden expansion into a bubble. The bubble coalesces with the neighboring bubble.

Video S9 Heat treatment of an exoskeletal droplet. Note during cooling, condensation of the cover slip temporarily obscures the view, but the droplet can still be tracked by its shadow.
